# The PARP inhibitor olaparib enhances the sensitivity of Ewing sarcoma to trabectedin

**DOI:** 10.18632/oncotarget.4303

**Published:** 2015-05-27

**Authors:** José Luis Ordóñez, Ana Teresa Amaral, Angel M. Carcaboso, David Herrero-Martín, María del Carmen García-Macías, Vicky Sevillano, Diego Alonso, Guillem Pascual-Pasto, Laura San-Segundo, Monica Vila-Ubach, Telmo Rodrigues, Susana Fraile, Cristina Teodosio, Agustín Mayo-Iscar, Miguel Aracil, Carlos María Galmarini, Oscar M. Tirado, Jaume Mora, Enrique de Álava

**Affiliations:** ^1^ Laboratory of Molecular Pathology, Instituto de Biomedicina de Sevilla (IBiS), Hospital Universitario Virgen del Rocio/CSIC/Universidad de Sevilla, Seville, Spain; ^2^ Developmental Tumor Biology Laboratory, Preclinical Therapeutics and Drug Delivery Research Program, Hospital Sant Joan de Deu Barcelona, Spain; ^3^ Sarcoma Research Group, Laboratori d'Oncología Molecular, Institut d'Investigació Biomèdica de Bellvitge (IDIBELL), L'Hospitalet de Llobregat, Barcelona, Spain; ^4^ Centro de Investigación del Cáncer, Instituto de Biología Molecular y Celular del Cáncer/Consejo Superior de Investigaciones Científicas, Universidad de Salamanca, Salamanca, Spain; ^5^ Statistics and Operations Research Department, University of Valladolid, Spain; ^6^ Cell Biology and Pharmacogenomics Department, Pharmamar, Madrid, Spain

**Keywords:** Ewing sarcoma, PARP inhibitor, trabectedin, DNA damage, PDX models

## Abstract

Recent preclinical evidence has suggested that Ewing Sarcoma (ES) bearing EWSR1-ETS fusions could be particularly sensitive to PARP inhibitors (PARPinh) in combination with DNA damage repair (DDR) agents. Trabectedin is an antitumoral agent that modulates EWSR1-FLI1 transcriptional functions, causing DNA damage. Interestingly, PARP1 is also a transcriptional regulator of EWSR1-FLI1, and PARPinh disrupts the DDR machinery. Thus, given the impact and apparent specificity of both agents with regard to the DNA damage/DDR system and EWSR1-FLI1 activity in ES, we decided to explore the activity of combining PARPinh and Trabectedin in *in vitro* and *in vivo* experiments. The combination of Olaparib and Trabectedin was found to be highly synergistic, inhibiting cell proliferation, inducing apoptosis, and the accumulation of G2/M. The drug combination also enhanced γH2AX intranuclear accumulation as a result of DNA damage induction, DNA fragmentation and global DDR deregulation, while EWSR1-FLI1 target expression remained unaffected. The effect of the drug combination was corroborated in a mouse xenograft model of ES and, more importantly, in two ES patient-derived xenograft (PDX) models in which the tumors showed complete regression. In conclusion, the combination of the two agents leads to a biologically significant deregulation of the DDR machinery that elicits relevant antitumor activity in preclinical models and might represent a promising therapeutic tool that should be further explored for translation to the clinical setting.

## INTRODUCTION

ES is an aggressive developmental mesenchymal tumor [1]. As a result of multimodal therapy, including strategies from traditional chemotherapeutic agents, radiotherapy, and surgery, the current cure rate of ES patients with localized disease is 70% [2, 3]. However, the survival rate for patients with relapsed, multifocal/metastatic disease is still less than 20%, mainly due to development of drug resistance. Recently, a retrospective study by our group revealed the prognostic value of chromosome 1 gain (1qG) in ES [4]. Within the up-regulated candidate genes identified in 1qG ES, a particularly interesting gene was found: poly (ADP-ribose) polymerase-1 (*PARP1*) [4]. Other studies have recently described that PARP1 plays an important role in tumors bearing ETS fusions, such as prostate cancer and ES [5, 6].

PARP proteins play a relevant role in the regulation of the cell cycle, apoptosis, genomic stability, chromatin remodeling, transcription, tumor growth and even the induction of chromosomal translocations. PARP1 has been shown to act as a transcriptional regulator of EWSR1-FLI1, which has triggered interest in testing PARPinh in ES [6, 7]. However, the best known activity of PARP proteins is related to the mechanisms of DDR, especially regarding the Base Excision repair (BER) pathway [8]. In brief, the BER is the main pathway responsible for repairing single-strand breaks (SSB) of DNA. In the absence of an effective BER mechanism, SSB accumulation results in Double-Strand Breaks (DSB), the most lethal form of DNA damage. Globally, when the DDR machinery fails this produces both an increase in the frequency of acquired mutations and genomic instability and both these determine the appearance, evolution and progression of tumors. In fact, an active DDR machinery is essential for the physiology of the cell, ensuring its survival, and is an important mechanism of resistance to cytotoxic agents. Accordingly, the inhibition of the DDR in tumor cells provides an excellent therapeutic opportunity [9]. Inhibitors of the DDR machinery have been used successfully against tumor cells in monotherapy or combined with other agents in order to sensitize tumor cells to the cytotoxic activity of other drugs. Within this large group of drugs, to date PARPinh represents one of the most promising subgroup of agents [8, 9]. In this sense, it has recently been reported that ES cells are defective in DNA breakage repair systems, being very sensitive to PARPinh in combination with DNA damaging-drugs [10].

Trabectedin is a marine-derived compound with proven antitumor activity against several types of neoplasms, including sarcomas, ovarian, and breast cancer, among others, by inducing DNA breaks. Clinical trials have shown that Trabectedin is particularly active in sarcomas bearing translocations, namely myxoid Liposarcoma (ML), ES and Synovial Sarcoma [11, 12]. In ES, *Grohar et al*. showed that, similarly to the previous observation in ML, Trabectedin interferes directly with the chimeric protein EWSR1-FLI1 [12]. In fact, treatment with Trabectedin specifically deregulates the transcriptional activity of the chimeric protein [12-14].

Given the impact and apparent specificity of PARPinh and Trabectedin with regard to the DNA damage/DDR system and EWSR1-FLI1 activity in ES, we decided to explore the effects of the combination through a series of *in vitro* and *in vivo* studies. We report that the combination of Trabectedin and Olaparib is highly synergistic in ES cell lines, inducing major DNA damage *in vitro* and *in vivo* and causing a clinically significant degree of tumor regression in PDX) models of ES.

## RESULTS

### ES cells are especially sensitive to olaparib, which induces G2/M accumulation independently of the 1q status

**Figure 1:**
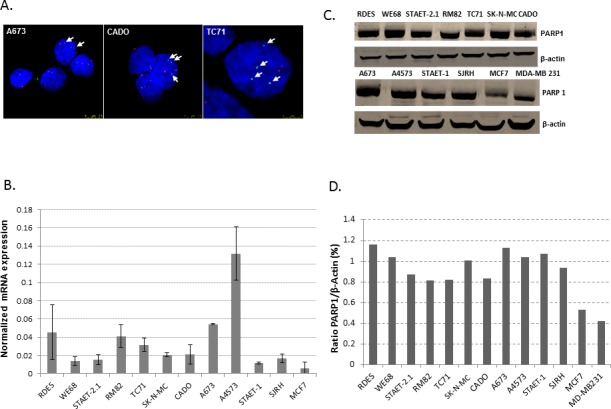
PARP1: gene status, mRNA and protein expression **A.** FISH of *PARP1.* ES cell lines previously described as 1qG showed extra-copies of *PARP1* (CADO and TC71 cell lines) whereas A673, described as 1qN, showed only two copies of *PARP1* by FISH. Green and red dots represent PARP1 gene copies and 1q centromeres, respectively. Arrows also indicate the green dots (PARP1 gene copies). **B.** q-RT-PCR shows that *PARP1* expression is heterogeneous among the ES cell lines studied. SJRH also express *PARP1* at similar levels to the ES cell lines. A4573 (1qG) and A673 (1qN) are the cell lines with the highest expression of *PARP1*. The MCF7 breast cancer cell line was used as a positive control. **C.** Western blotting showed that ES cell lines express PARP1 under normal conditions. Here, breast cancer cell lines (MCF7 and MD-MB 23) were used as positive controls. **D.** Quantification of PARP1 protein levels with respect to β-actin expression using Image J software.

Initially, we checked the status of *PARP1* in a panel of ES cell lines (Supplementary Table S1) using a home-made fluorescence *in situ* hybridization (FISH) probe specific for *PARP1*. We observed that ES cell lines previously described as 1qG in fact showed an amplification of *PARP1* by FISH (Figure 1A). cDNA analysis showed that all ES cell lines express *PARP1* with different mRNA levels (Figure 1B). Also, using a specific antibody for PARP1, by Western-blot we observed that all cell lines studied expressed PARP1 at similar levels, independently of the 1qG status, (Figure 1C-1D).

**Figure 2:**
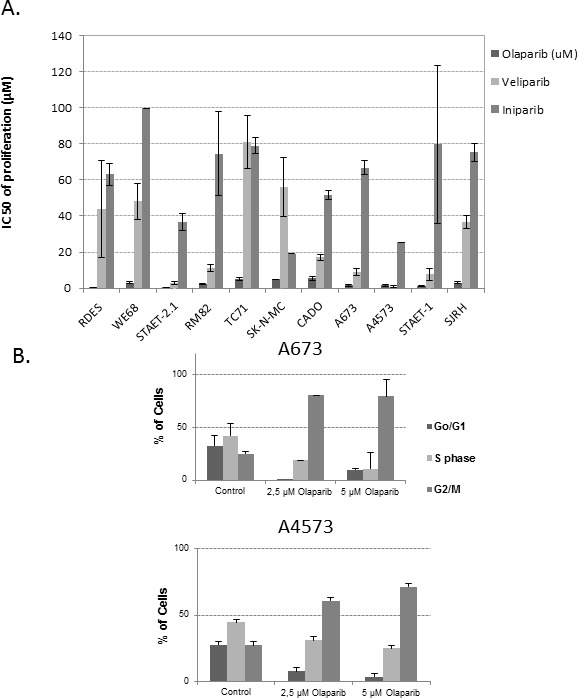
PARPinh activity: proliferation and cell cycle analysis **A.** IC50 of proliferation after 72 hours of drug exposure. ES cell lines and the SJRH cell line were more sensitive to Olaparib than to the other agents **B.**. Cell cycle analysis on A673 (1qN) and A4573 (1qG) after treatment with two different concentrations of Olaparib.

We studied the sensitivity of ES cell lines to a group of PARPinh, including Olaparib, Veliparib and Iniparib. Olaparib was more active in inhibiting proliferation than the other two drugs assayed, with lower IC_50_ levels at 72 hours of exposure (high nM-low μM range, with a median of 1.995 ± 0.46μM). Veliparib was the second most effective agent, with IC_50_ levels of proliferation in the μM range, with a median of 14.14±2.75μM (approximately 7 fold higher than Olaparib). Finally, Iniparib was the least effective agent, showing IC_50_ levels also in the μM range but with a median of 74.95 ± 5.02μM (approximately 38-fold higher than Olaparib) (Figure 2A). Interestingly, we observed that after 72 hours of exposure to Olaparib IC_50_ levels were higher than those obtained after 6 days of treatment (Supplementary Figure S1A). Given that *PARP1* is located on chromosome 1q, and in view of our previous results describing some ES tumors and cell lines with 1qG, we searched for a correlation between the status of 1q [Gained or Normal (N)] and Olaparib sensitivity (Supplementary Figure S1B). We observed a trend towards a higher sensitivity of 1qG cell lines to Olaparib, but it was not statistically significant, probably due to the low number of cell lines analyzed (Mann Whitney U test, *p* > 0,05). The correlation between the status of *p53* (wild-type or mutated) and the sensitivity to Olaparib was not statistically significant either (Mann Whitney U test, *p* > 0.05) (Supplementary Figure S1C). We also studied the effects of Olaparib on the cell cycle profile using two ES cell lines, A673 (1qN) and A4573 (1qG) (Figure 2B). Both cell lines showed G2/M accumulation after treatment, even at low doses of Olaparib (Figure 2B).

### The combination of olaparib and trabectedin is highly synergistic in ES cell lines

Having observed that Olaparib was much more cytotoxic than the other PARPinh, we studied the effects of the combination of Olaparib with Trabectedin. ES cell lines (*n* = 10) were exposed to different combinations of both agents at a constant ratio of 1:20.000 (Trabectedin:Olaparib) for 72 hours and Combination Indices (CIs) were determined according to [15, 16]. Interestingly, synergistic effects were observed in all but two cell lines (Table 1). We then studied the effects of this drug combination on apoptosis induction via caspase -3 and -7 activation after 48 hours of drug exposure as well as cell cycle effects after 24 hours of treatment in two cell lines, RM82 and TC71. We observed that the drug combination (250pM of Trabectedin and 5μM of Olaparib) increased the apoptotic rate in the TC71 cell line (Supplementary Figure S2A). In agreement with this, we observed cleaved PARP accumulation after treatment with the combination, especially at 24 hours, in the TC71 cell line (Supplementary Figure S2B). Furthermore a G2/M accumulation and the elimination of the G0/G1 phase were also observed after treatment with the drug combination (Supplementary Figure S2C). In the RM82 cell line, treatment with the drug combination only induced mild apoptosis (Supplementary Figure S2A). By contrast, no effects on cell migration after 72 hours of drug exposure were observed (Supplementary Figure S3).

**Table 1:** Proliferation assays show that the combination of Olaparib and Trabectedin is synergistic in all ES cell lines with the exception of A4573 and WE68, where the CIs are almost additive (values close to 1)

Cell lines	CI	Effects
RDES	0.73	Synergism
WE68	0.92	Nearly additive
STAET-2.1	0.49	Synergism
RM82	0.69	Synergism
TC71	0.4	Synergism
SK-N-MC	0.008	Synergism
CADO	0.4	Synergism
A673	0.77	Synergism
A4573	0.93	Nearly additive
STAET-1	0.64	Synergism
SJRH	0.11	Synergism

### The combination of olaparib and trabectedin enhances DNA damage while EWSR1-FLI1 activity is not deregulated

**Figure 3:**
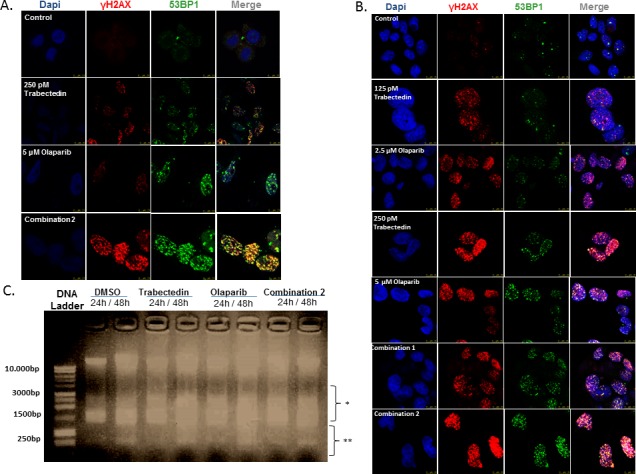
DNA damage induction studies (A and B) Images represent data obtained in the study of induction of DSB evaluated by the presence of intranuclear γH2AX and 53BP1 foci after treatment with Trabectedin and/or Olaparib in the TC71 cell line. **A.** Short-term DNA damage induction (8 hours of drug exposure). The combination of Trabectedin and Olaparib led to an increased presence of pH2AX and 53BP1 foci in the nucleus. **B.** Long-term DNA damage induction (24 hours of drug exposure). **C.** Image represents data obtained in the fragmentation assays after 24 – 48 hours of drug exposure. Although DNA fragmentation was also observed in the control (DMSO), Trabectedin and/or Olaparib induced higher DNA fragmentation. Here, DSB-induced DNA fragmentation can be seen by the presence of a smear just below the intact DNA (represented by *), whereas the smaller fragments (represented by **) are produced exclusively by apoptosis induction. In all cases, combination 1 refers to 125pM Trabectedin and 2.5μM Olaparib; combination 2 refers to 250pM Trabectedin and 5μM. Olaparib.

DNA damage induction was evaluated by testing for the presence of intranuclear foci of γH2AX and 53BP1 after short (8 hours) and long (24 hours) drug exposures. Initially, cells were treated with Trabectedin (250pM) and/or Olaparib (5μM) for 8 hours. Trabectedin induced a stronger DSB accumulation than that induced by Olaparib alone (Student's *t*-test, *p* < 0.05). Nevertheless, the combination of both agents resulted in a clear enhancement of DSB formation (Figure 3A and Supplementary Figure S4). After 24 hours of drug exposure, the drug combinations resulted in cumulative effects of DNA damage through the induction of DSB, even at the lowest drug concentrations used (125pM of Trabectedin and 2.5 μM) (Figure 3B). Interestingly, the combination did not affect EWSR1-FLI1 transcriptional activity (Supplementary Figure S5A).

Moreover, DNA fragmentation assays revealed that both drugs were able to generate enough damage to induce DNA fragmentation (Figure 3C and Supplementary Figure S5B).

### Olaparib and/or trabectedin induce deregulation of the DDR machinery

**Figure 4:**
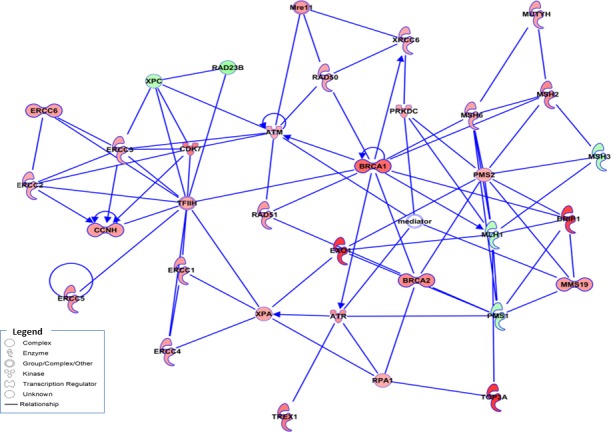
Expression changes in DNA damage repair genes in ES cells from the TC71 cell line Schematic representation of gene interactions in cells treated with the combination of Olaparib (5μM) and Trabectedin (250pM), obtained after performing IPA. Over-expressed genes are highlighted in red, and the down-regulated genes are highlighted in green. The central genes involved in HR machinery are highlighted in color, such as *BRAC1*, *EXO1*, *RAD51* and *BRCA2,* among others.

After demonstrating that the combination of Olaparib and Trabectedin induced DNA damage by DSB accumulation, we next analyzed the regulation of the DDR machinery in TC71 cells. To this end, we used low-density arrays (DDR PCR Array; PAHS-042Z, Qiagen). After 16.5 hours of treatment with Olaparib (5μM) and/or Trabectedin (250pM), RNA was extracted and the changes in the mRNA levels regarding DNA damage induction and DDR mechanisms were analyzed. Treatment with Trabectedin caused an overexpression of homologuous recombination (HR)-related genes such as *BRCA1, BRCA2, RAD18, RAD52* and *BRIP1* (*BRCA1/2*-associated molecule) as well as *POLD3* (DNA polymerase delta subunit 3); *NEIL3* (nei endonuclease VIII-like 3) involved in the BER pathway; R*FC1* (replication factor C), which is involved in GC-NER DSB repair; *OGG1*; *XRCC1*, involved in DNA- and protein-binding and strand-break correction, and *UNG*, a DNA glycosylase involved in the BER pathway (Supplementary Tables S2, S3 and Supplementary Figure S6). Treatment with Olaparib induced the expression of the HR-related genes *EXO1, RAD54L, RAD51B* and *RAD18*, together with *BRIP1, RFC1, PARP3, XRCC, POLD3* and *PARP3* (Supplementary Tables S2, S3 and Supplementary Figure S7). The global deregulation of the DNA damage and repair machinery was clearly observed with the combined treatment, where the HR genes *RAD54L, EXO1, PARP2, BRCA1, BRCA2, RAD18, RAD50, RAD51* and *RPA1* were overexpressed (Supplementary Table S2, S3 and S4). Changes in expression in this array were highly correlated with DSB repair by the NER pathway (p value: 1.02E-23), the NHEJ pathway (p-value: 1,81E-23), the HR pathway (p-value: 4,41E-18) and the related BRCA1 and BRCA2 pathways (p-values 4,52E-18 and 2,24E-17) (Supplementary Table S4). Ingenuity Pathway Analysis (IPA) discriminated the most significant upregulated and downregulated molecules involved in the major DNA damage pathways. The combination treatment induced a stronger deregulation of the molecules involved in these pathways (Figure 4).

### The drug combination reduces tumor growth dramatically in an *in vivo* model

In view of the results obtained in the *in vitro* study, we decided to test the antitumor effects of the Trabectedin and Olaparib combination in a NOD/SCID mouse xenograft model of ES. Xenografts were generated by subcutaneous injection of TC71 cells. Animals were randomized in 5 groups and treated for three weeks: 1) Control group: drug vehicles; 2) Trabectedin group: Trabectedin alone (0.15mg/Kg IV once a week five times per week); 3) Olaparib group: Olaparib (100mg/Kg BID IP five times per week); 4) Combination1 group: Olaparib alone (100mg/Kg BID IP five times per week) for 1 week, and Olaparib plus Trabectedin (0.15mg/Kg IV once a week) for the second and third week; 5) Combination 2 group: Olaparib (100mg/Kg BID IP) plus Trabectedin (0.15mg/Kg IV once a week). After three weeks of treatment, the mice were sacrificed and tumors were extracted, weighed, and evaluated by histopathology.

**Figure 5:**
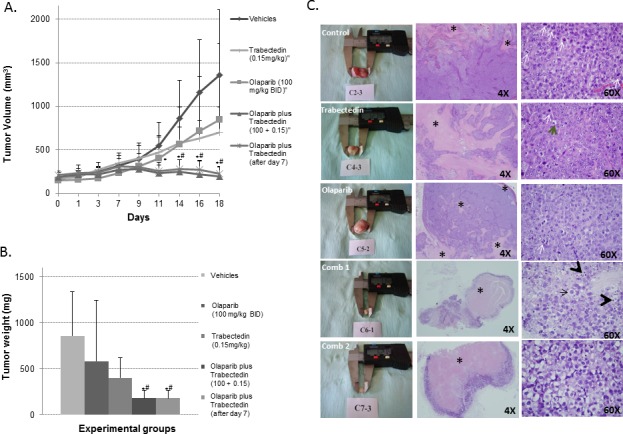
*In vivo* model using the TC71 cell line **A.** Graphic representing the evolution of tumor growth in the different treatment groups. Tumor growth was clearly halted by the combination of Olaparib and Trabectedin (Combination2) and when Trabectedin began to be administered one week later (Combination 1). **B.** Graphic representation of mean tumor weights at the end of the study (18 days after treatment began to be administered) in each of the treatment groups. Tumor weight was clearly reduced, especially in the Combination 1 and 2 groups. **C.** Representative pictures showing (in columns from left to right) tumors extracted and 4X and 60X H&E representative areas of the tumors from each treatment group respectively. In the second column some necrotic areas are indicated among areas of living cells (*). In the third column, the tumors in the control group show characteristic small round cells with a round nucleus, regular chromatin, a clear nucleolus, scant and rare cytoplasm and a high number of mitotic figures (white arrows). By contrast, the tumors in the treated groups, and, especially in the two combination groups, showed degenerate cells (black arrow), with a vacuolated and degenerate cytoplasm and apoptotic figures (black arrowhead). Comb 1 refers to Combination 1. Comb 2 refers to Combination 2. (* or ^#^ mean *p* < 0.05; *, Comb 1 or Comb 2 *vs*. control; ^#^Comb 1 or comb 2 vs. Olaparib alone or Trabectedin alone).

The evolution of tumor volume after randomization (see Material and Methods section) and tumor weight at the end of the study (18 days after the treatments had started), shown in Figure 5A and 5B respectively, were evaluated in all groups. Although Trabectedin and Olaparib alone were able to reduce tumor volume as compared with the control groups, these differences were not statistically significant (Mann-Whitney *U-test*, *p* > 0.05). However, the combination of both drugs dramatically reduced tumor growth (Mann-Whitney *U-test p* < 0.05). In fact, the tumors were smaller at the end of the study than they were at the moment of randomization, before treatment.

Histopathological analysis of representative tumors from each group of mice revealed significant morphological changes. The drug-induced morphological changes were characterized by the presence of ES cells with conspicuous nucleoli, granular chromatin, an enlarged cytoplasm and vacuoles (Figure 5C). Necrosis was observed in the animals of all groups, although it was significantly more extensive in the animals treated with the combination of drugs (*t*-test, *p* < 0.05) (Figure 5C and Supplementary Figure S8A). The necrotic/viable tumor ratio was much higher in the treated groups (Supplementary Figure S8A, S8B). No signs of toxicity were observed with the combination treatment.

### The drug combination increases DNA damage induction and DDR activation in the *in vivo* model

A representative number of tumor areas from tumors of all groups in the *in vivo* study were evaluated by Immunoflorescence (IF)/ Immunohistochemstry (IHC) in order to assess the effects of the treatments. To quantify the findings, we used the Ariol Image analysis system (Olympus). The following parameters were studied: apoptosis (fragmentation-late apoptosis and PARP cleavage) (TUNEL assay and cleaved PARP); proliferation (Ki67), DNA damage induction (γH2AX) and DDR activation (BRCA2).

**Figure 6:**
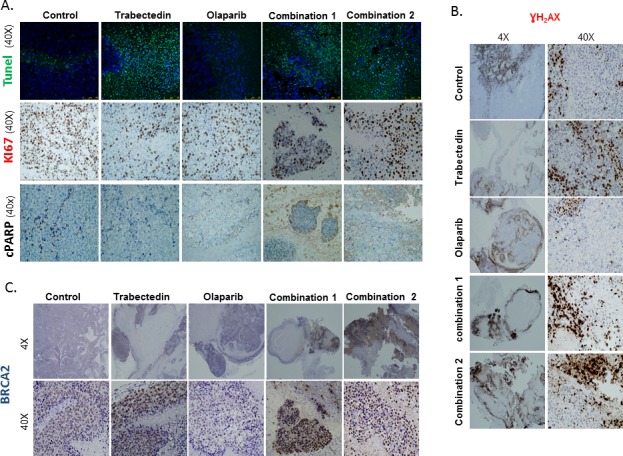
Immunofluorescence and Immunohistochemical analysis of tumor xenografts: Apoptosis, proliferation and DNA damage induction **A.** The images show a higher number of Ki67 positive cells in the control group as compared to tumors treated with Trabectedin alone or with the combinations of Olaparib and Trabectedin (central panel). In the TUNEL assay, cell staining was mostly observed in tumors from the Trabectedin group and from the combination groups (upper panel). PARP cleavage only revealed a slight staining in the Olaparib group. **B.** The images show intranuclear γH2AX staining in tumors from all the groups but it is more extensive and intense mainly in non-necrotic areas of the tumors from the Olaparib and Trabectedin combinations. **C.** The images show an intense, focal BRCA2 staining in tumors from the Trabectedin and the combination groups. Very little expression was observed in the Olaparib group. Combination1 refers to 100mg/kg Olaparib and 0.15mg/kg Trabectedin, which started to be administered after day 7. Combination2 refers to Olaparib and 0.15mg/kg.Trabectedin.

The proliferation index was higher in the controls as compared with all treated groups, and this was statistically significant (*p* < 0.05) (Figure 6, central panel A and Supplementary Figure S8B). These results are in agreement with the data from the TUNEL assays, where late apoptosis due to DNA fragmentation was mostly observed in representative tumor areas from animals treated with Trabectedin alone or in tumors from animals treated with the combination of drugs (Figure 6, upper panel A). PARP cleavage was observed in tumors from the Olaparib group (Figure 6, lower panel A).

Interestingly, DNA damage induction as assessed by pH2AX staining was much higher in animals from both combination groups (mean Combination 1: 60.975%, mean Combination 2 group: 68.09%) as compared with controls (mean 0.85%); Trabectedin (mean 11.13%) and Olaparib (mean 1.04%) (*p* < 0.05) (Figure 6B and Supplementary Figure S8C, S9). This observation correlated well with the overexpression of the HR effector molecule, BRCA2. Treatment with the combination of Olaparib and Trabectedin resulted in a much more intense and diffuse staining of BRCA2, especially in combination 2 (Combination1: mean 42.03%; Combination 2: mean 76.79% *vs*. mean control group 11.9%) (Supplementary Figure S8D). Additionally, BRCA2 expression was diffuse and intense in the case of tumors from the Trabectedin group (mean 63.84%) (Figure 5C and Supplementary Figure S8D, S10).

### The trabectedin-olaparib combination induces complete tumor regression in PDX models

**Figure 7:**
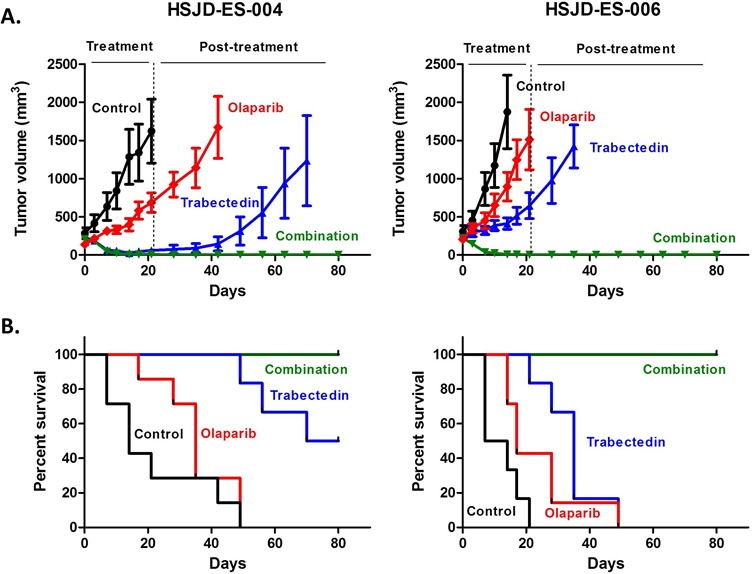
Tumor growth **A.** and animal survival **B.** of HSJD-ES-004 and HSJD-ES-006 PDX models upon single-agent and combination therapies. Olaparib and Trabectedin in combination induced complete tumor regression **A.** and improved overall survival **B.** in both PDX models as compared with single treatments and controls.

Because studies in PDX models are likely to be more predictive of patient response to treatment than conventional cell lines [17], we performed survival studies in two ES PDX models (HSJD-ES-004 and HSJD-ES-006) established from patient biopsies at the Sant Joan de Déu Hospital (HSJD, Barcelona, Spain). The clinical characteristics of the patients and the molecular details of both tumor models are shown in Table 2. The tumor response at the end of treatment is shown in Table 3. The most remarkable finding was that the Olaparib-Trabectedin combination achieved complete response (CR) in 100% of the tumors of both PDX models. In contrast, Olaparib treatment alone led to progressive disease (PD) in 100% of the tumors. Trabectedin alone achieved CR in 88% of the HSJD-ES-004 tumors and CR in 78% of the HSJD-ES-006 tumors. CRs in monotherapy were transient in most animals and did not persist until the end of the survival study (Figure 7A). In the HSJD-ES-004 model, the median survival of the controls was 14 days, as compared to 35 days for the Olaparib group (*p* = 0.192), and 75 days for the Trabectedin group (*p* = 0.0 01). In the HSJD-ES-006 model, the median survival of the controls was 11 days, as compared to 17 days for the Olaparib group (*p* < 0.05), and 35 days for the Trabectedin group (*p* = 0.001). The combination therapy extended survival significantly, up to a value greater than the evaluation period (80 days), when compared with the control mice (*p* < 0.05 for both models; Figure 7B).

**Table 2:** Details of the ES PDX models

Model code	Source of biopsy^1^	Age at biopsy	Primary tumor	Age at diagnosis	Metastasis at diagnosis	Demography	Fusion gene	STAG2 mutation	p53 mutation	Patient status
HSJD-ES-004^2^	Relapse. Metastasis in the mediastinum	18 y	T12 vertebral body	10 y	No	Male, white	EWSR1-FLI1	negative	negative	NED^3^
HSJD-ES-006	Relapse. Metastasis in the lung	14 y	Fibula	12 y	No	Male, white	EWSR1-FLI1	negative	negative	AWD^4^

1 Tumor tissue was collected with informed consent under an Institutional Review Board-approved protocol.

2 Further details on this PDX model are published in [21], coded SJDES022-R.

3 No evidence of disease.

4 Alive with disease.

**Table 3:** ES PDX models: response to treatment (day 21)

PDX Model	Treatment	Tumors (n)	Response^1^			
			CR	PR	SD	PD
HSJD-ES-004	Control	10	0	0	0	10
	Olaparib	9	0	0	0	9
	Trabectedin	8	7	0	0	1
	Combination	8	8	0	0	0
HSJD-ES-006	Control	10	0	0	0	10
	Olaparib	10	0	0	0	10
	Trabectedin	9	1	1	0	7
	Combination	9	9	0	0	0

1 CR: Complete response (CR) was defined, as previously described in [34], as the disappearance of measurable tumor mass (<0.10 cm^3^) at the end of treatment (day 21); PR: Partial response (PR) was defined as a tumor volume regression ≥50% at the end of treatment (day 21) but with a measurable tumor size (≥0.10 cm^3^); SD: Stable disease (SD) was defined as <50% regression from the initial volume and a ≤25% increase in initial volume at the end of treatment (day 21). PD: Progressive disease (PD) was defined as <50% regression from the initial volume and a >25% increase in the initial volume at the end of treatment (day 21).

## DISCUSSION

Trabectedin induces DNA damage and cellular death *in vitro* and interrupts EWSR1-FLI1-dependent transcription [12]. Additionally, ES cells are sensitive to PARPinh, which disrupt the DDR system of tumor cells [7, 10]. Furthermore, PARP1, the main PARPinh target, also acts as a transcriptional regulator of EWSR1-FLI1 [6]. Accordingly, we formulated the hypothesis that the combination of Trabectedin with a PARPinh could interfere with the DDR system and might be able to inhibit EWSR1-FLI1, and could therefore be of interest as a therapeutic strategy. The results obtained in the present study support the clinical development of a combination of PARPinh with Trabectedin in ES.

The relationship between PARP1 and ES has been documented for many years [18]. However, knowledge of the more specific and relevant role of this protein in ES is much more recent [6, 7]. Brenner *et al*. described that PARP1 interacts physically with EWSR1-FLI1 fusion, acting in a positive feed-back loop and hence regulating its own expression [6]. In a retrospective study, our group has reported that 1qG was of prognostic significance in ES [4] and in the present study we show that 1qG is strongly linked to the upregulation of *PARP1*, *among* other genes [4].

The mechanism of action of PARPinh in cancer cells appears to be mainly related to the induction of DNA damage by the formation of irreparable SSB. These drugs are used in monotherapy in tumors with baseline molecular inactivation in some of the DDR system components, such as *BRCA1/BRCA2* [19, 20]. The use of PARPinh in monotherapy to treat patients with breast cancer defective in *BRCA1* and/or *BRCA2* is a clear example of what is known as synthetic lethality [19, 20]. So far, there is no evidence of *BRCA1/BRCA2* mutations in ES [21]. However, the use of PARPinh has been expanded to tumors harboring ETS translocations, such as prostate cancer and ES [5, 6]. In ES in particular, sensitivity to PARPinh seems to be related to EWSR1-FLI1 transcriptional activity [6, 7].

Olaparib is the drug chosen in most studies addressing ES [6, 7, 22]. In the present study, we decided to test the efficacy of three different PARPinh, all of them extensively studied in solid tumors. Olaparib and Veliparib have a similar mechanism of action in that they compete with nicotinamide adenine dinucleotide (NAD) to bind the catalytically active site of the PARP enzyme. By contrast, Iniparib (BSI-201) does not compete with the active site of the PARP enzyme but induces an irreversible modification of PARP1 and stimulates a PARP1-protease [23]. Although Iniparib was tested in a phase III study in triple-negative breast cancer patients, its efficacy and specificity have recently been questioned [24]. According to the results of our study, Iniparib is by far the least active of the three PARPinh tested. By contrast, Olaparib was the most active compound tested against ES cell lines, with an IC_50_ for proliferation ranging from the high nanomolar to the low micromolar range, very similar to the data published by other groups [7]. Interestingly, and in contrast to previous work [7], Olaparib was seen to be not only a potent cytotoxic agent after 3 days of drug exposure, but its action lasted for at least 6 days, when the IC_50_ underwent a significant decrease in all the cell lines assayed. However, in the *in vivo* study, Olaparib alone was only partially effective in terms of tumor shrinkage. This is also in accordance with previous studies, in which Olaparib was not able to halt tumor growth [6]. In fact, a recent clinical trial of Olaparib alone in relapsed/refractory ES patients was withdrawn due to the lack of objective responses (Revised in [1]).

Since PARPinh are not only used in monotherapy but also in combination with radiotherapy and other chemotherapeutic agents that cause DNA damage, we decided to combine Olaparib with Trabectedin. Interestingly, the groups of Grohar and Burdach have recently suggested combining Trabectedin with drugs that further increase DNA damage and diminish EWSR1-FLI1 transcriptional activity. According to the hypothesis of Grohar and Burdach, the repression of the downstream targets of an oncogene increases cancer cell sensitivity; such repression can be specifically addressed by combined precision chemotherapy, increasing the therapeutic index of the combined agents and overcoming resistance to highly selective targeted therapies [14, 25]. Grohar *et al*. demonstrated that the combination of Trabectedin and Irinotecan in ES exhibits synergistic activity, confirming the molecular precision treatment concept [14, 25]. This concept could also be applied to the combination of PARPinh and Trabectedin. In fact, PARPinh, and in particular Olaparib, are involved in DNA damage, and the knockdown of PARP1 in ES cell lines disrupts the expression of known EWSR1-FLI1 target genes [6]. Grohar et al. found a decreased expression of known EWSR1-FLI1 up-regulated target genes, such as *NR0B1*, and very little expression of down-regulated target genes after treatment with Trabectedin at 10nM for 12 hours in TC71 and TC32 ES cells [12]. Our results showed that the combination of Trabectedin and Olaparib was highly synergistic, increasing apoptotic activity and arresting the cell cycle at the G2/M phases for ES, but that it did not deregulate EWSR1-FLI1 target expression in the TC71 cell line. This antitumor activity was also observed *in vivo*, where the drug combination reduced tumor growth dramatically, even when Trabectedin was administered one week later than Olaparib. Furthermore, we observed that although both agents alone induced DNA damage through an accumulation of γH2AX foci and deregulation of the DDR pathways *in vitro*, the combination significantly increased this phenomenon. This drug combination actively deregulated the major DDR pathways, without changing EWSR1-FLI1 target expression. The effects of the drug combination *in vivo* confirmed the *in vitro* data, the accumulation of γH2AX foci increasing with the overexpression of BRCA2. Most importantly, a complete and maintained regression of tumors for 80 days due to the combination therapy was observed in ES PDX models. To our knowledge, this is the first time PDX mouse models have been reported in ES. These models are renewable tumor models engrafted in mice from fresh human ES samples without prior *in vitro* growth. PDX models have been shown to be biologically stable and to accurately reflect patient tumor biology and treatment response in other tumor types [26, 27]. Thus, the results of our study could be considered sufficient to justify the design of a clinical trial using the combination of Olaparib and Trabectedin in ES. Other studies have combined Olaparib with drugs/strategies inducing DNA damage, namely Temozolomide and radiotherapy [6, 22]. In both cases the combination regimen showed a reduction in tumor volume in comparison with the controls. However, DNA damage induction and the DDR activation machinery were not studied in depth. Here, we observed not only that the combination is active both *in vitro* and *in vivo* but also that it induces a deregulation of the DDR pathways. A recent study has shown that ES cell lines are defective in DDR and when the PARPinh BMN-673 was combined with Irinotecan and Temozolomide, 88% of complete responses were achieved in an orthotopic model of ES [10]. In our study, complete responses were achieved with the combination of Olaparib and Trabectedin in 100% of mice from two ES PDX models studied.

In conclusion, to the best of our knowledge this is the first time that the combination of a PARPinh and Trabectedin has been reported in ES. The combination could be a useful therapeutic strategy in ES that should be further explored.

## MATERIALS AND METHODS

### Cell culture

Ten ES cell lines (RDES, WE68, STAET-2.1, RM82, TC71, SK-N-MC, CADO, A673, A4573, STAET-1) and the SJRH Rhabdomyosarcoma cell line were grown in RPMI supplemented with 10% Fetal Bovine Serum (FBS) and 1% antibiotic (P/S), except that A673 ES cell line was grown in DMEM supplemented with 10% FBS (Gibco, Invitrogen). All ES cell lines were obtained from the FP6-funded Eurobonet consortium cell line panel, which is maintained and regularly checked and characterized [4]. All ES cell lines and SJRH were grown on gelatin-coated plates in an incubator at 37ºC and 5% CO2. MCF7 and MDA-MB-231 breast cancer cell lines were cultured in DMEM medium supplemented with 10% Fetal Bovine Serum (FBS) and 1% antibiotic (P/S) (Gibco, Invitrogen) and 1% glutamine (Gibco, Invitrogen). All cells were free of mycoplasma, as screened at least once a month with the MycoAlert® Mycoplasma Detection Kit (Lonza).

### Drugs

The following PARPinh were used: Olaparib (LC Laboratories), Veliparib (AxonMEDCHEM), Iniparib (LC Laboratories). All drugs were prepared at the appropriate stocking concentration in DMSO (Sigma) and stored at −20°C until use. Trabectedin was kindly provided by PharmaMar (Madrid, Spain).

### Proliferation assays

Briefly, cells were counted by Trypan blue and seeded 24 hour prior to treatment in complete medium on 24-well culture plates or 96-well culture plates. Treatments were added later and cells were incubated for 72 hours. The medium was then removed and cells were incubated for 1h with a 1:10 MTT solution (Sigma-Aldrich). The medium was later removed and replaced by 500μl of DMSO (Merck). Absorbance was then read on an Infinite^®^ F500 Tecan plate reader (Tecan) at 570nm. The number of cells seeded for each assay depended on the type of cell line and its growth rate. To define drug-drug interactions (in terms of synergism, additivity, or antagonism), the CI was calculated according to the Chou-Talalay method [15, 16], using CalcuSyn software Version 2.0 (Biosoft). Synergy levels can be broadly divided into: < 0.1 very strong synergism; 0.1-0.90, synergism (ranging from strong synergism to slight synergism), and 0.90-1.10, nearly additive to additive.

### Detection of caspase 3 and 7 activities

The activity of caspases 3 and 7 was determined in a Caspase Glo (Promega) luminescence assay. Initially, cells were seeded in white-walled 96-well plates for 24 hours and then treated with the drug for 24 hours. After incubation with the drug, 100μl of a mixture of caspase Glo buffer and substrate, prepared at the time at RT, was added per well. Plates were incubated for 1 hour and luminescence was read on an Infinite^®^ F500 Tecan plate reader (Tecan). Caspase 3 and 7 activity was calculated by subtracting the blank value (well with medium but no cells) and was normalized to the controls (untreated cells).

### PARP1 probe design and FISH

Two specific and independent *PARP1* locus probes (BAC Clones RPII-964L17 and RPII-831N20) labeled with Spectrum green-dUTP (green signal) (Vysis) were made. Probes were labeled as previously described [28, 29]. Briefly, isolated BAC DNA was labeled with Spectrum red-dUTP (Vysis) by nick translation (Vysis) and purified after adding 10μg of COT-1. A commercially available (CEP 1) probe (Vysis) labeled with spectrum red (red signal) was also used. To check the specificity of the home-made probes, co-hybridizations using both commercial CEP1 and *PARP1* specific probes were performed on peripheral blood cells in metaphase. FISH analysis was performed on cell lines fixed in methanol-acetic acid. A volume of 10μl of the diluted probes was applied to the slides. The slide was covered with a glass coverslip and sealed with rubber cement. Using a Hybrite machine (Vysis), denaturation was performed at 75°C for 5 minutes and hybridization was carried out at 37°C for at least 16 hours. After removing the coverslips, post-hybridization washes were done at 46ºC in 2XSSC, 50% formamide, for 5 minutes and stained with DAPI (6-diamidino-2-phenylindole) and mounted with Vectashield H-1000 medium (Vector). Digital images were obtained using a Zeiss Axioplan2 epi-fluorescence microscope (Carl Zeiss) equipped with a digital camera (ORCA-ER-1394, Hamamatsu Photonics KK). In all cases, one hundred nuclei were counted [28, 30].

### Protein extraction and western blotting

Protein extraction and western blotting were done as previously described [31, 32]. Briefly, primary antibodies were used: pH2Ax (Cell Signaling); PARP (Cell signaling), Calnexin (Santa-Cruz) and Actin (Sigma) were incubated overnight at 4ºC in TBS-T 0.5% BSA (Sigma). Secondary anti-Rb-Cy3 and anti-Ms-Cy5 antibodies were incubated for 1 hour at RT and the images were recovered using Odissey software. The quantification of protein levels was accomplished using Image J software (National Institutes of Health).

### Immunofluorescence

Cells were seeded on slide covers placed (one slide cover per well) on 96-well plates pretreated with 1% gelatin/ MilliQ H_2_O (Sigma) for 24 hours in complete medium. The medium was removed and the cells were incubated in medium with drug/DMSO (Sigma) for 6 hours (short exposure) or 24 hours (long exposure). After treatment, cells were fixed in ice-cold methanol (Sigma) for 15 minutes, washed twice, permeabilized with 0.1% Triton X-100 for 30 minutes, and blocked in PBS with 2% BSA (Sigma) for another 30 minutes. The pH_2_AX primary antibody (Cell Signaling) was then incubated overnight, and after several washes the Cy3 secondary antibody (Jackson ImmunoResearch) was incubated for 1 hour. After 15 minutes of washes, the primary anti-53BP1 antibody (Abcam) was incubated for 3 hours at RT followed by several washes, and the Cy5 secondary antibody was incubated for 1 hour at RT. After several washes, DAPI at 1mg/ml in PBS was used to stain nuclei, applying it for 15 minutes. Finally, slide covers were mounted using Mowiol (Sigma) and were observed under a Leica microscope using Leica software. pH2AX fluorescence was quantified using Image J software, where at least 50 nuclei were analyzed using the following formula: Corrected Total Cell Fluorescence (CTCF) = Integrated Density (IT)- (Area of selected cell-Mean of fluorescence of background readings). The number of intracellular foci of pH2AX was measured by counting independent intranuclear foci of at least 50 nuclei.

### Real time RT-PCR

RNA was extracted from all cell lines studied using a Qiagen mini kit (Qiagen). Following Qiagen's instructions, 2μg of high quality RNA was reverse-transcribed (Qiagen). Primers for the *PARP1* gene were designed using primer 3 software (MIT, Cambridge, MA, USA), and quantitative reverse transcription-PCR (qRT-PCR) was performed as described earlier using SYBR Green [32]. qRT-PCR was performed under universal cycling conditions on an ABI 7300HT instrument (Applied Biosystems), using commercially available TaqMan probes: *PARP1* (HS00242302-m1); *NR0B1* (HS03043658-m1); *NKX2-2* (HS00159616-m1); *TGFβR2* (HS00234253-m1); *CAV1* (HS00971716_m1) and Mastermix (all from Life Technologies). Cycle threshold (*C*_T_) values were normalized to *Beta Actin* (*ACTB*). Experiments were performed at least twice and in triplicate. The relative expression level was calculated as described previously [33].

### Low-density microarrays

RNA was extracted from the TC71 cell line, using a Qiagen mini kit (Qiagen). Following Qiagen's instructions, 0.5μg of high-quality RNA were reverse-transcribed and hybridized on a Human DNA Repair PCR Array (Qiagen). The following protocol was set in an iQ5 BioRad thermo cycler (Bio-Rad): 10 minutes cycle at 95ºC for Taq Polymerase activation, followed by 40 cycles of 15 seconds at 95ºC and 1 minute at 60ºC to perform fluorescence data acquisition. Data were analyzed using the online software http://www.sabiosciences.com/pcrarraydataanalysis.php. Pathway analysis was performed using IPA (Ingenuity systems), as previously described [32].

### Cell cycle

Propidium iodide (PI) staining was used to analyze cell cycle changes by Fluorescence-Activated Cell Sorting (FACS). Cells were seeded in 6 well-plates and incubated with the appropriate drug for 24 hours. PI staining was performed as described elsewhere [31, 32]. Cells were acquired (20,000 events) with a FACSort Cytometer (Becton Dickinson). Analysis of the results was performed using Infinicyt (Cytognos), Paint-a-Gate (Becton Dickinson) and ModFit software (Verity Software House).

### Migration assays

These assays were performed by wound healing, where 5 × 10^4^ cells were seeded in 24-well plates and when confluence reached 90%, a wound was performed with a pipette tip from one side to the other. Medium with drug was added and incubated for 8/24/48 and/or 72 hours. After drug exposure, cells were washed, stained with 1% crystal violet (Sigma-Aldrich) in 2% ethanol (Sigma-Aldrich), and observed with a Leica Microscope (Leica Microsystems), where pictures were obtained.

### *In vivo* study

4-5 week-old female NOD/*scid* mice were used. Induction of tumour xenografts was performed by subcutaneous injection of cell suspensions, containing 5×10^6^ of TC71 live cells in 0.2 ml of 1:1 cellular medium/Matrigel matrix (Becton Dickinson) into the right flank of the mice. This study followed the Spanish and European Union guidelines for animal experimentation (RD 1201/05, RD 53/2013 and 86/609/CEE, respectively). Mice were randomized into control and treatment groups (*n* = 10) one week after the tumor started to be measurable (10 days after injection: day 0 of treatment). Drugs were administered in the following vehicles: 0.9% NaCl in sterile water, vehicle of Trabectedin and/or 2-hydroxyl-propyl-B cyclodextrin 10% in PBS plus 10% DMSO (Sigma-Aldrich), vehicle of Olaparib. Mice with tumor volumes greater than 1.5 cm^3^ were excluded from the analysis. Tumors were measured every 2-3 days with a caliper and diameters were recorded. Tumor volumes were calculated using the formula: *a*^2^*b/*2, where *a* and *b* are the 2 maximum diameters. Mice were sacrificed by anaesthetic overdosing 4 weeks after cell injection, and tumors, liver and kidneys were collected for histopathological analyses. All experimental manipulations with mice were performed under sterile conditions in a laminar flow hood.

### *In vivo* preclinical testing in ES PDX models

NOD/SCID mice (Harlan, Barcelona, Spain) were used for initial engraftment and further passages were performed in athymic nude mice (Harlan). For the survival studies, nude mice bearing subcutaneous 200-500 mm^3^ tumors in both flanks were randomized into 4 groups of 5 mice. One group received 100 mg/kg Olaparib twice daily (5 days on, 2 off) IP for 2 weeks; a second group was treated with 0.15 mg/kg Trabectedin (the MTD) IV on days 1 and 8; a third group received the combination of Olaparib and Trabectedin under the same regimens; and a fourth group acted as controls. Mice were sacrificed when the tumor diameter reached 1.5 cm^3^ and the study was finalized at day 80 post-treatment.

To study the activity of the different regimens, we evaluated tumor response at the end of treatment (day 21) and animal survival until the end of the study (day 80). Tumor response was evaluated as described elsewhere [34]. Animal survival was defined as the time interval between the initial date of treatment and the date on which the threshold 1.5 cm^3^ tumor volume was reached.

### Histopathology and immunohistochemistry

Excised tumors, kidney and livers were sampled just after sacrifice and representative areas were a) formalin-fixed (24 hours) (Millipore) and paraffin-embedded and (b) snap-frozen in OCT and stored at 80ºC as previously described [32]. Tissue sections 2μM thick were stained with hematoxilin & eosin and prepared for IHC. Two experienced pathologists (MCGM and EDA) observed the samples under a Leica microscope (Leica Microsystems). IHC was performed as previously described [32] using the following primary antibodies: anti-pH2AX (Millipore); anti-cleaved PARP (cell signaling); anti-Ki67 (Millipore) and anti-BRCA2 (Millipore). TUNEL assays (Roche) were performed to detect DNA fragmentation and late apoptosis. To quantify the extent of necrosis and the IHC findings, the Dotslide analysis program (Olympus) and the Ariol Image analysis system (Olympus) were used respectively.

### Statistical analysis and bio software

Analysis of the low-density micro arrays was performed using the online software http://www.sabiosciences.com/pcrarraydataanalysis.php. IC50 values were calculated from linear transformation of the dose-response curves. Differences among groups were analyzed with the Mann–Whitney *U-*test*,* Student's t test or ANOVA. Median survivals were calculated using Kaplan-Meier curves and the log-rank test was used for statistical comparisons between each treatment group and the control group (Graphpad Prism 5 software). Significance was considered when *p* < 0.05.

## SUPPLEMENTARY FIGURES AND TABLES


