# Correction: The PARP inhibitor olaparib enhances the sensitivity of Ewing sarcoma to trabectedin

**DOI:** 10.18632/oncotarget.18610

**Published:** 2017-06-27

**Authors:** José Luis Ordóñez, Ana Teresa Amaral, Angel M. Carcaboso, David Herrero-Martín, María del Carmen García-Macías, Vicky Sevillano, Diego Alonso, Guillem Pascual-Pasto, Laura San-Segundo, Monica Vila-Ubach, Telmo Rodrigues, Susana Fraile, Cristina Teodosio, Agustín Mayo-Iscar, Miguel Aracil, Carlos María Galmarini, Oscar M. Tirado, Jaume Mora, Enrique de Álava

**Present**: The published funding information is incomplete.

**Correct:** Additional funding information is given below.

**Funding**

This project has received funding from the European Union’s Seventh Framework Programme for research, technological development and demonstration under grant agreement nr 602856 (EEC) and nr 278742 (EUROSARC).

Original article: Oncotarget. 2015; 6:18875-90. doi: 10.18632/oncotarget.4303

