# Supplementary material for: The PARP inhibitor olaparib enhances the sensitivity of Ewing sarcoma to trabectedin
Source: Oncotarget. 2015 May 27;6(22):18875–90. doi: 10.18632/oncotarget.4303 (PMC4662461; doi:10.18632/oncotarget.4303)
Supplement: Supplementary file 1 [file oncotarget-06-18875-s001.pdf]

The PARP inhibitor olaparib enhances the sensitivity of Ewing sarcoma to trabectedin

Supplementary Material

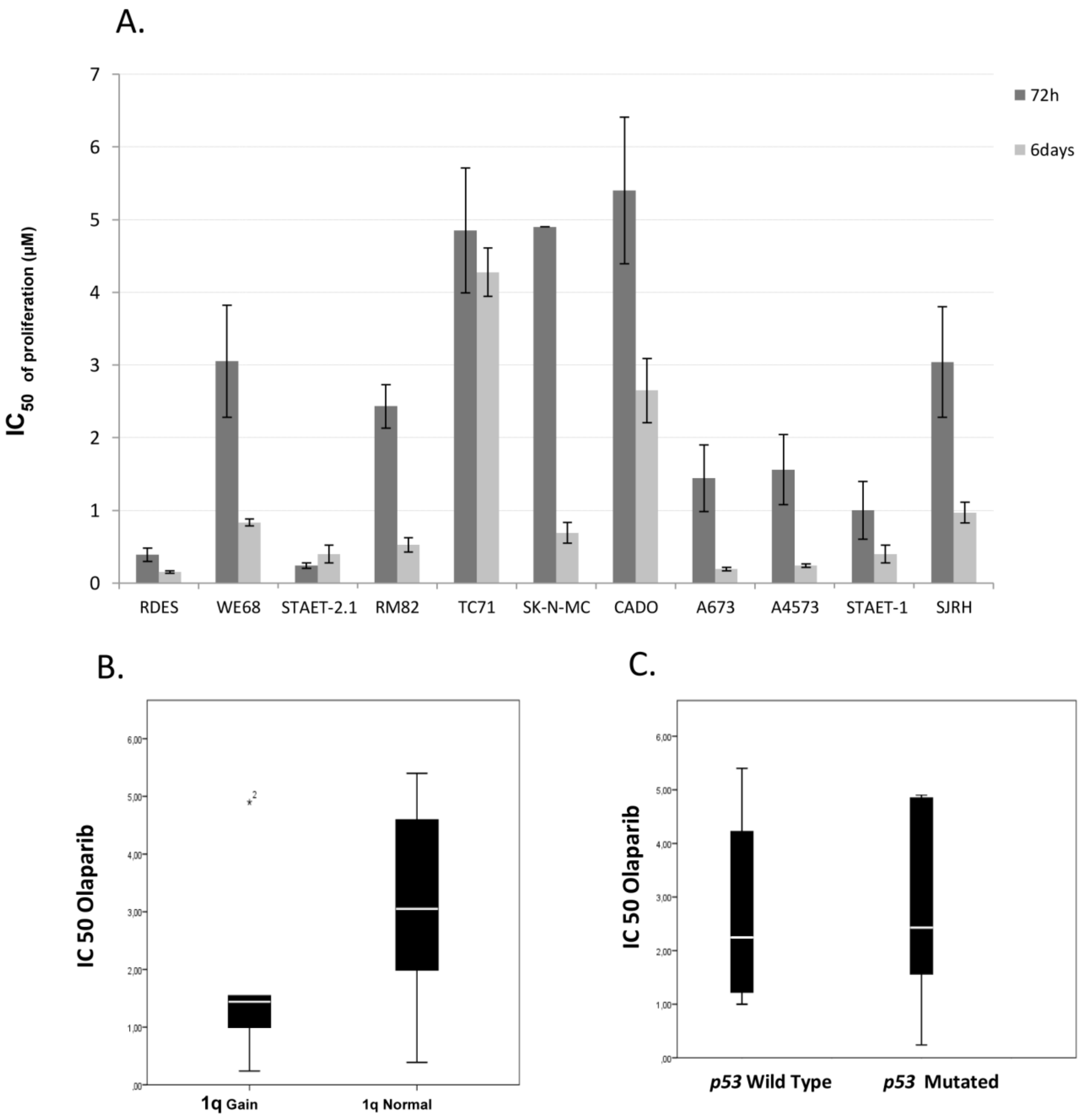

Supplementary Figure S1: (A) IC50 of proliferation after treatment with Olaparib: 72 hours versus 6 days. (B) Grafic representation of IC50 of ES cell lines after treatment with Olaparib with regard to ES cell lines1q status. (C) Grafic representation of IC50 of ES cell lines with regard to their p53 status.

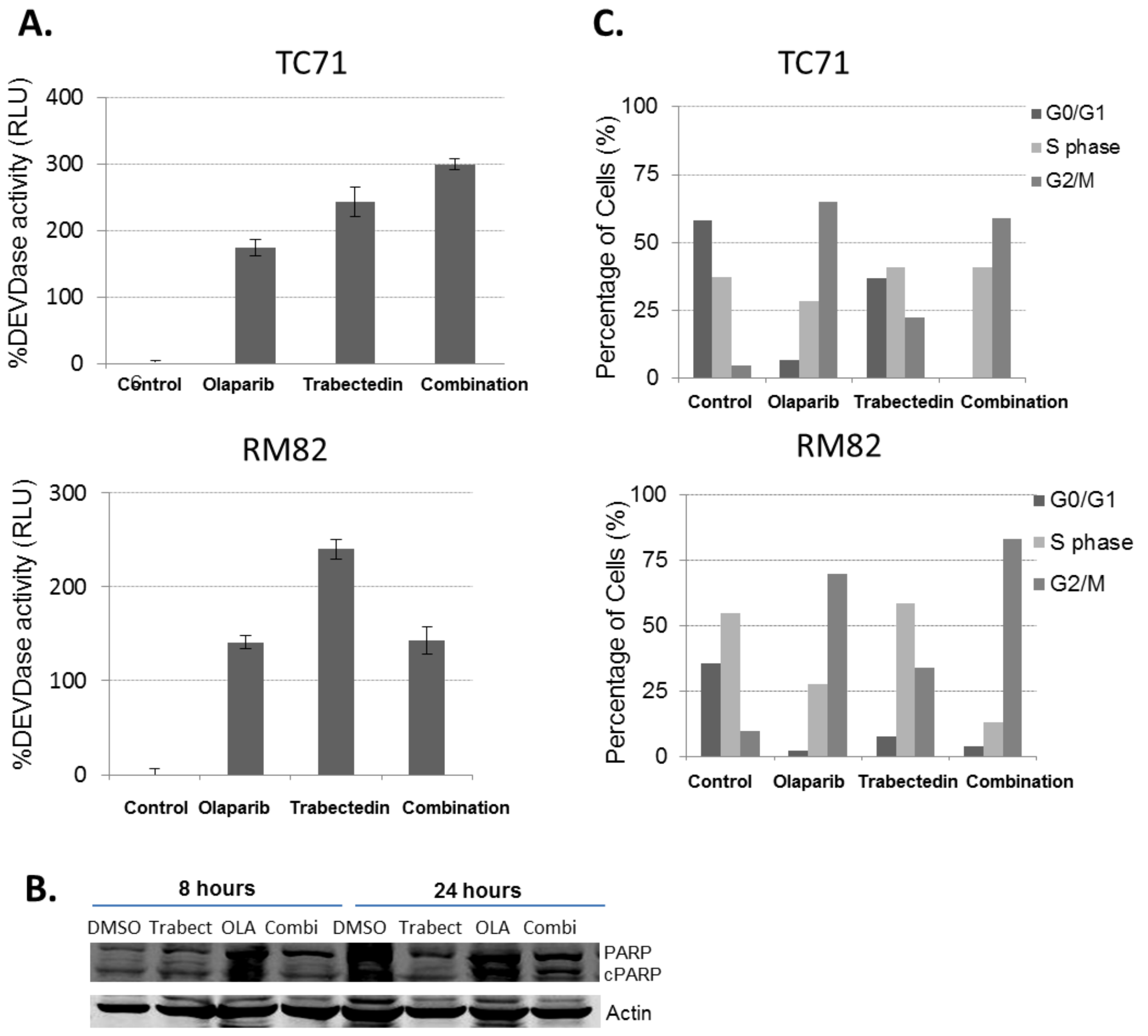

Supplementary Figure S2: (A). Graphics represent data obtained in the apoptosis assay determined by caspase 3 and 7 DEVDase activity. RLU stands for Relative Light Units. Treatment with Olaparib increased apoptosis as compared to the controls (141% in RM82 and 174% in TC71 cell lines). Treatment with Trabectedin elicited a higher apoptosis induction (240% and 242% in RM82 and TC71 cell lines respectively). The combination of Olaparib and Trabectedin in TC71 cells induced an increase in apoptosis as compared with the effects induced by drugs alone (299%). (B) PARP cleavage was evaluated by Western Blot in TC71 cells after treatment with the drugs alone/combined in short (8 hours) and longer (24 hours) administration. The combination induced an increase in PARP cleavage, especially when compared to Trabectedin. (C) Graphics representing data from cell cycle analysis after 24 hours of treatment in the RM82 and TC71 cell lines. Trabectedin induced S-phase accumulation, whereas Olaparib induced strong G2/M accumulation. The combination induced accumulation in the S-phase and G2/M and a significant decrease or even the abrogation of G0/G1 phase. In all cases, combination refers to 250pM Trabectedin and 5µM Olaparib. OLA refers to Olaparib.

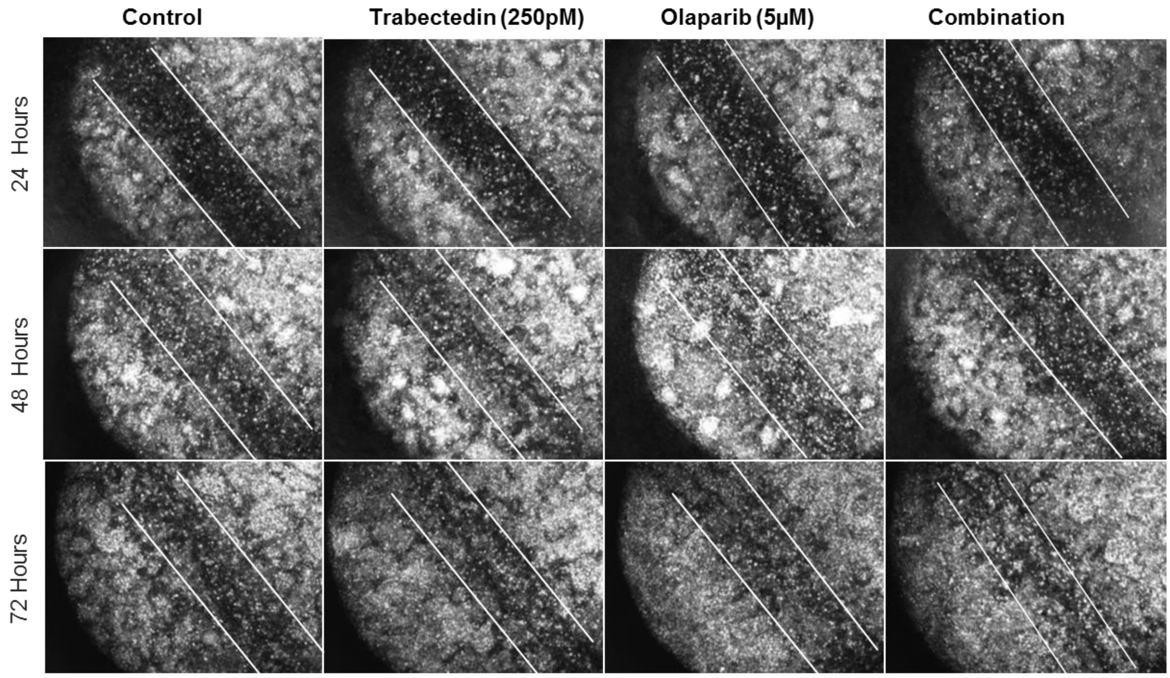

Supplementary Figure S3: Migration assays performed by wound healing in the RM82 cell line after treatment. No differences in cell migration were observed after drug exposure at 24, 48, and 72 hours as compared to the controls. Combination stands for 250pM Trabectedin and 5μM Olaparib.

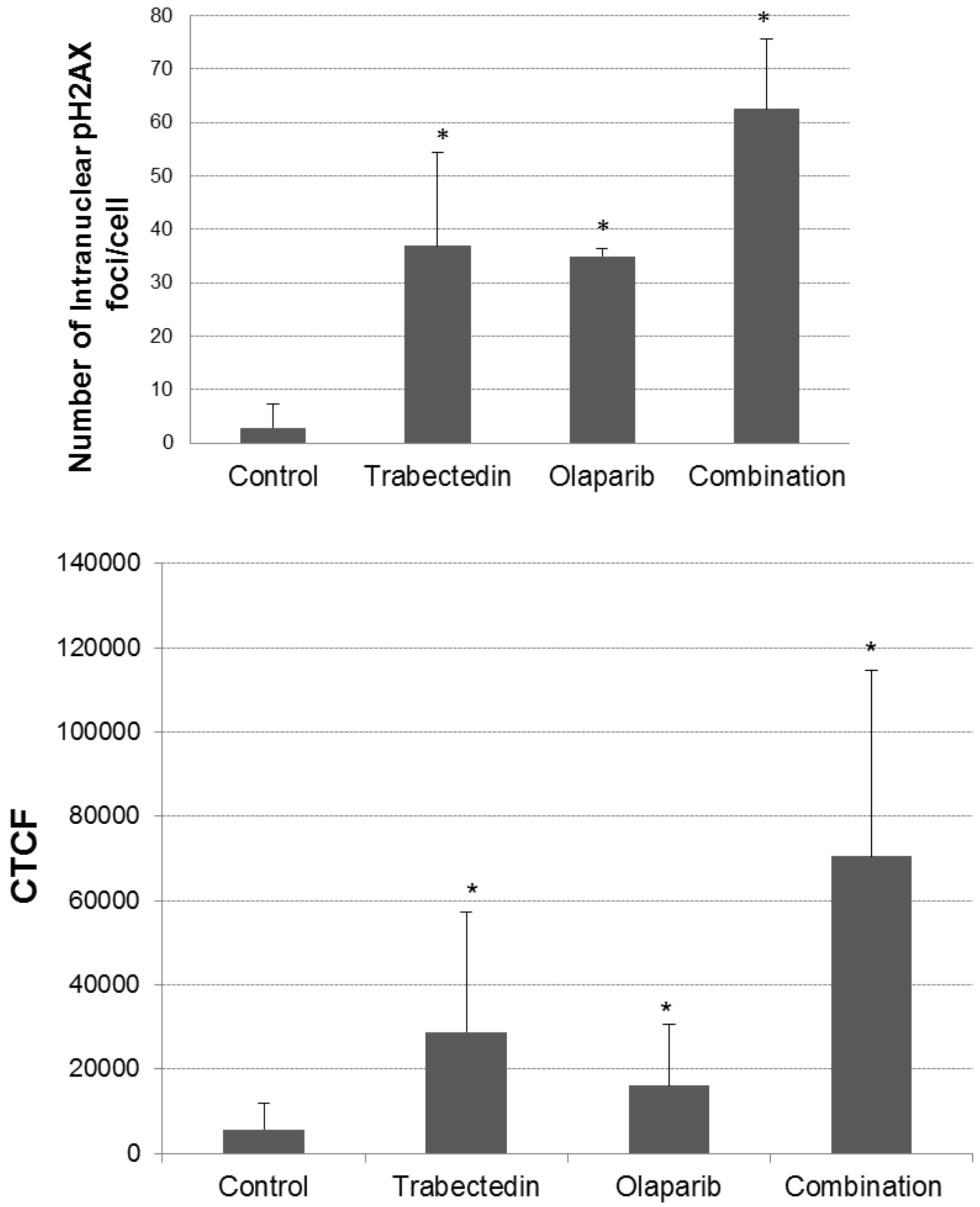

Supplementary Figure S4: Quantification of p-H2AX foci in the TC71 cell line after treatment. The number of pH2AX foci per cell and the total number of foci as measured by CTCF were higher in cells treated with the combination of drugs. CTCF stands for Corrected Total Cell Fluorescence, normalized to background subtraction. Trabectedin (250pM), Olaparib (5µM). Combination refers to 250pM Trabectedin and 5µM Olaparib. * $p<0,05$ Student's T-Test.

A.

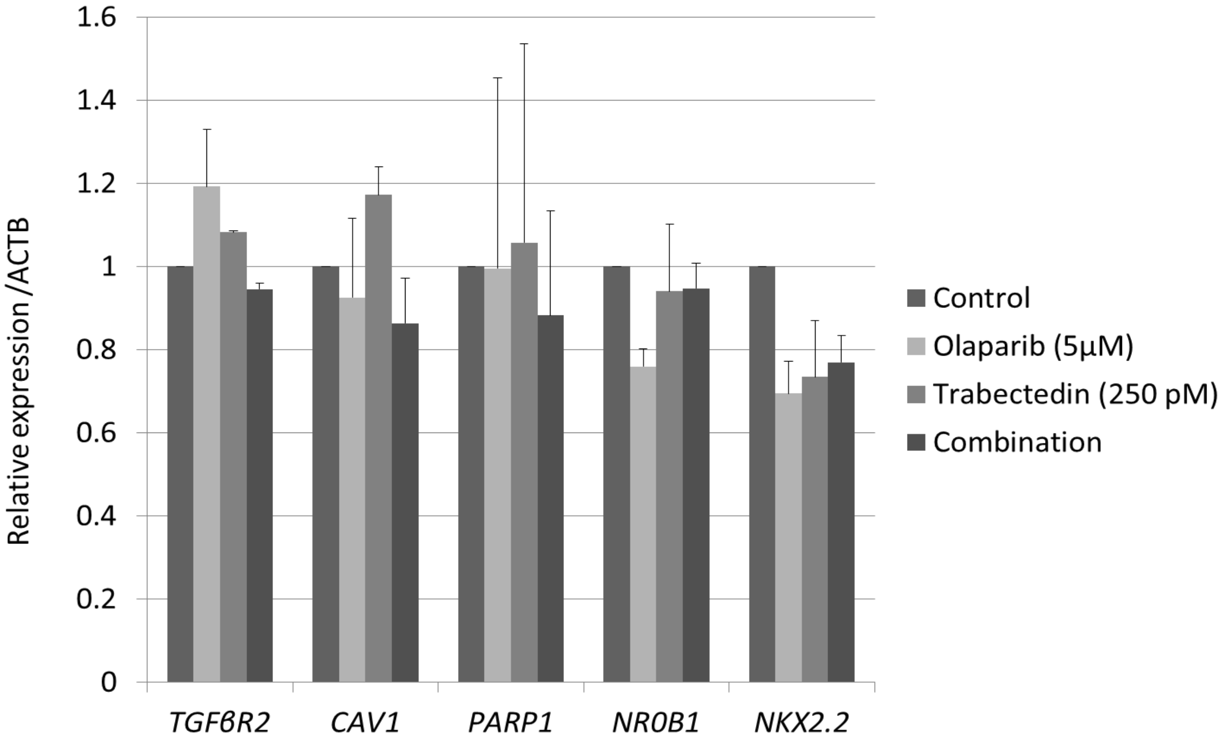

B.

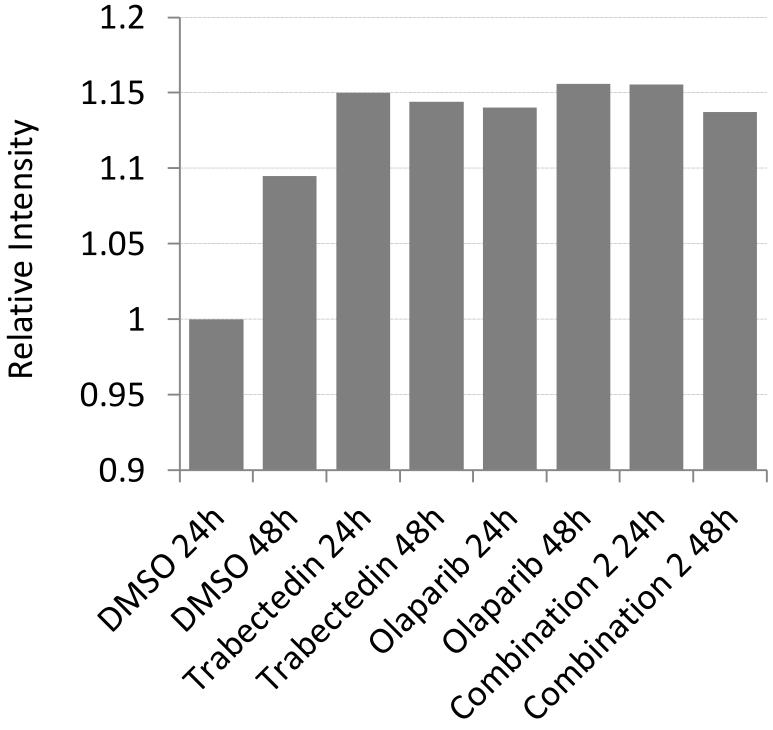

Supplementary figure S5: A) EWSR1-FLI1 target expression after treatments in the TC71 cell line. The expression of EWSR1-FLI1 targets was not affected after treatment with the drug combination. Only *NKX2.2*, a known upregulated indirect target of EWSR1-FLI, was slightly down-regulated after exposure to the combination of drugs. Combination refers to 250pM Trabectedin and 5µM Olaparib. B) Quantification of DNA damage induction shown in figure 3 C using Image J software. Relative intensity is shown as compared to control condition (DMSO).

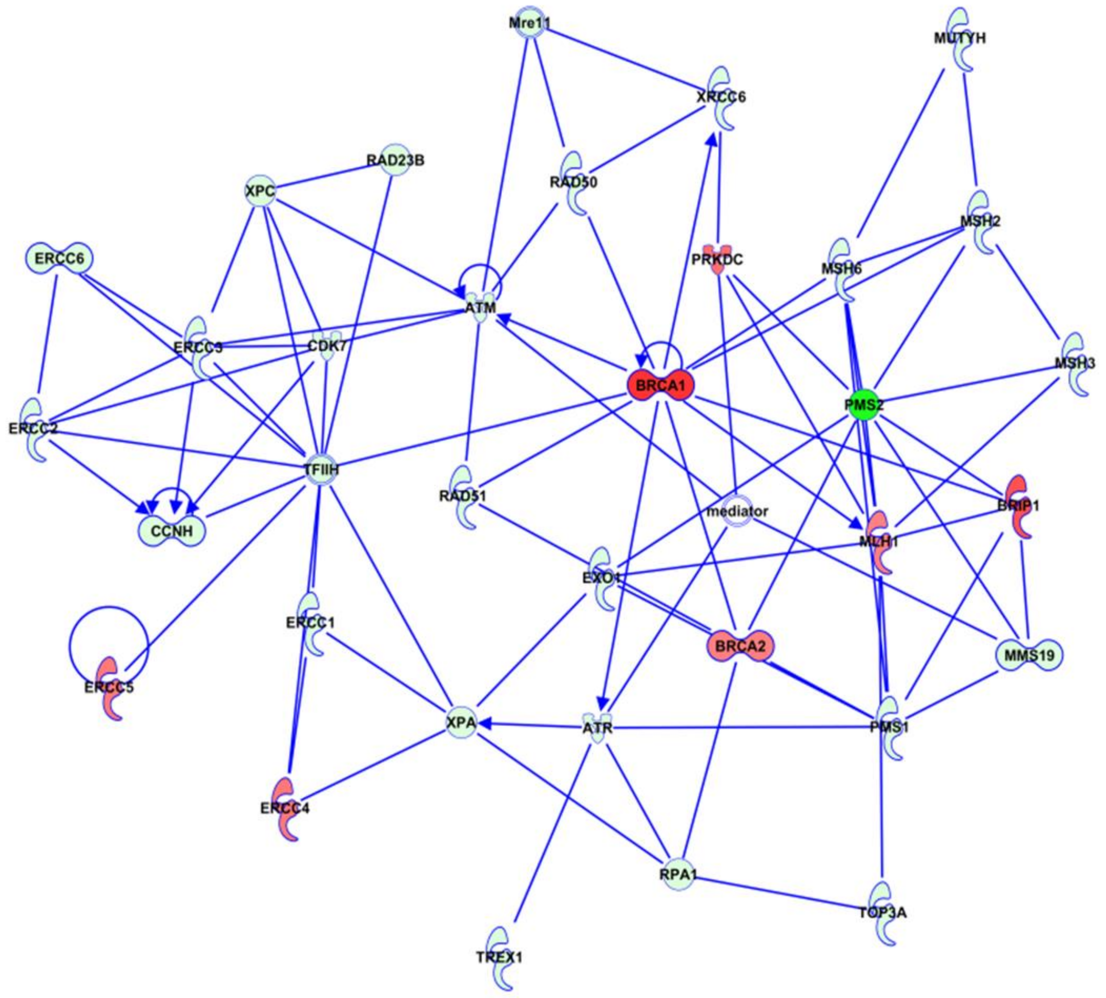

Molecules	Exp. Value
BRCA1	1.644
POLD3	1.394
BRIP1	1.382
RAD18	1.347
NEIL3	1.346
RFC1	1.247
OGG1	1.229
RAD52	1.205
XRCC1	1.166
UNG	1.160

Molecules	Exp. Value
PMS2	-625.000
RAD51B	-44.444
RAD51	-10.846
MSH5	-8.795
TOP3B	-8.532
PMS1	-8.019
LIG1	-5.952
XPA	-4.653
FEN1	-4.137
XRCC6	-4.050

Supplementary Figure S6: IPA analysis in the condition of Trabectedin alone.

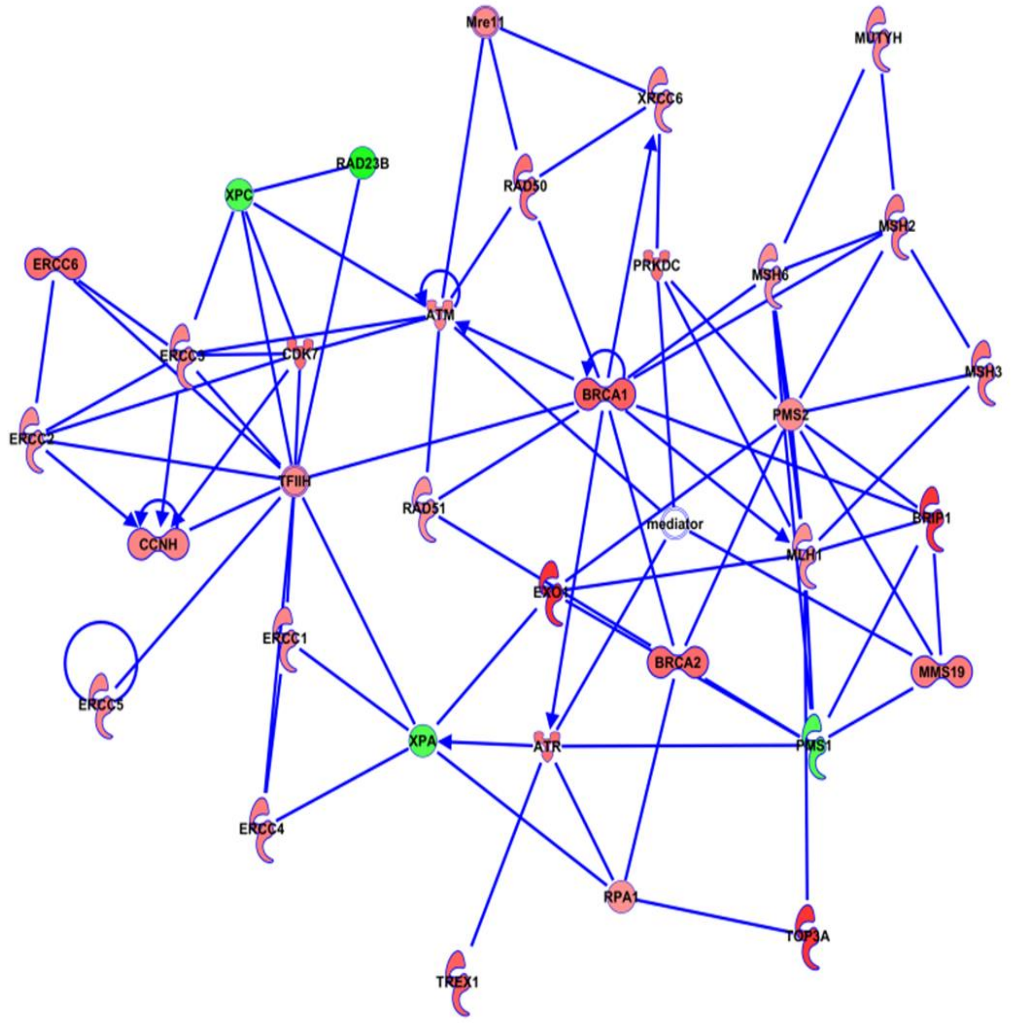

Molecules	Exp. Value
EXO1	1.927
BRIP1	1.904
TOP3A	1.903
RAD54L	1.886
RFC1	1.700
PARP3	1.625
RAD51B	1.613
XRCC1	1.609
POLD3	1.597
RAD18	1.550

Molecules	Exp. Value
RAD23B	-1.302
NEIL2	-1.089
NEIL1	-1.046
DMC1	-1.044
ATXN3	-1.044
NTHL1	-1.037
XPC	-1.027
PARP1	-1.017
XPA	-1.005
PMS1	-1.003

Supplementary Figure S7: IPA analysis in the condition of Olaparib alone.

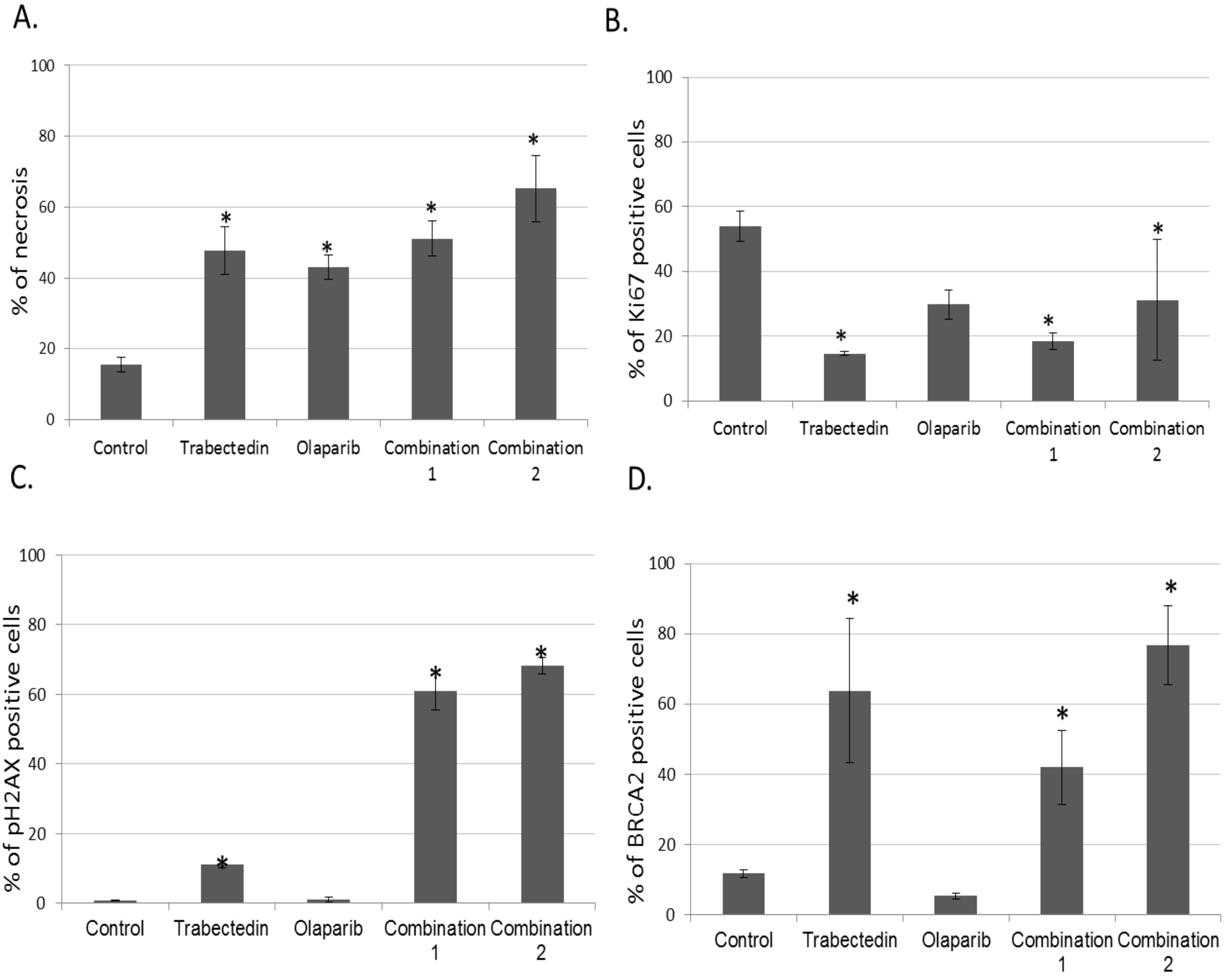

Supplementary Figure S8: Quantification of the histological findings found in tumors treated in the *in vivo* study. (A) Tumors treated with Trabectedin or Olaparib, and especially with the combination of both drugs, showed a higher % of necrosis. (B) Tumors treated with Trabectedin or combinations showed a smaller % of proliferating cells. (C) Tumors treated with combinations showed a higher number of pH2AX-positive cells. (D) Tumors treated with combinations or Trabectedin showed a higher number of BRCA2-positive cells. Student's T-test $*p<0.05$. Combination 1 refers to 100mg/kg Olaparib and 0.15mg/kg Trabectedin, which started to be administered 7 days after the start of administration of Olaparib. Combination 2 refers to 100mg/Kg Olaparib and 0.15mg/kg Trabectedin. The Dotslide analysis program (Olympus) was used to quantify the extent of necrosis. The Ariol Image analysis system (Olympus) was used to quantify pH2AX-, BRCA2- and Ki67-positive cells.

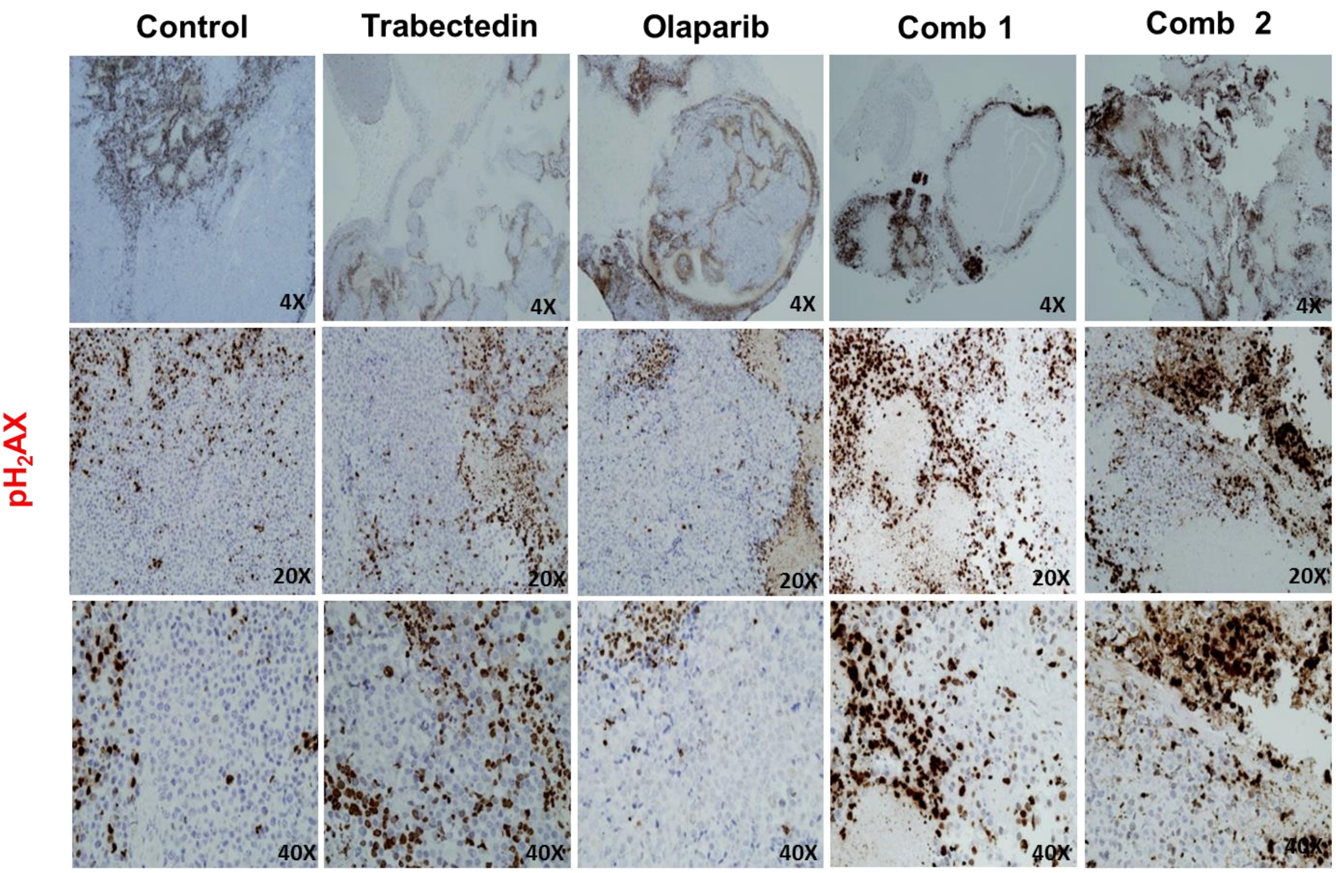

Supplementary Figure S9: γH2AX immunostaining performed in tumor sections derived from the *in vivo* study. Comb 1 refers to Combination1. Comb 2 refers to Combination2

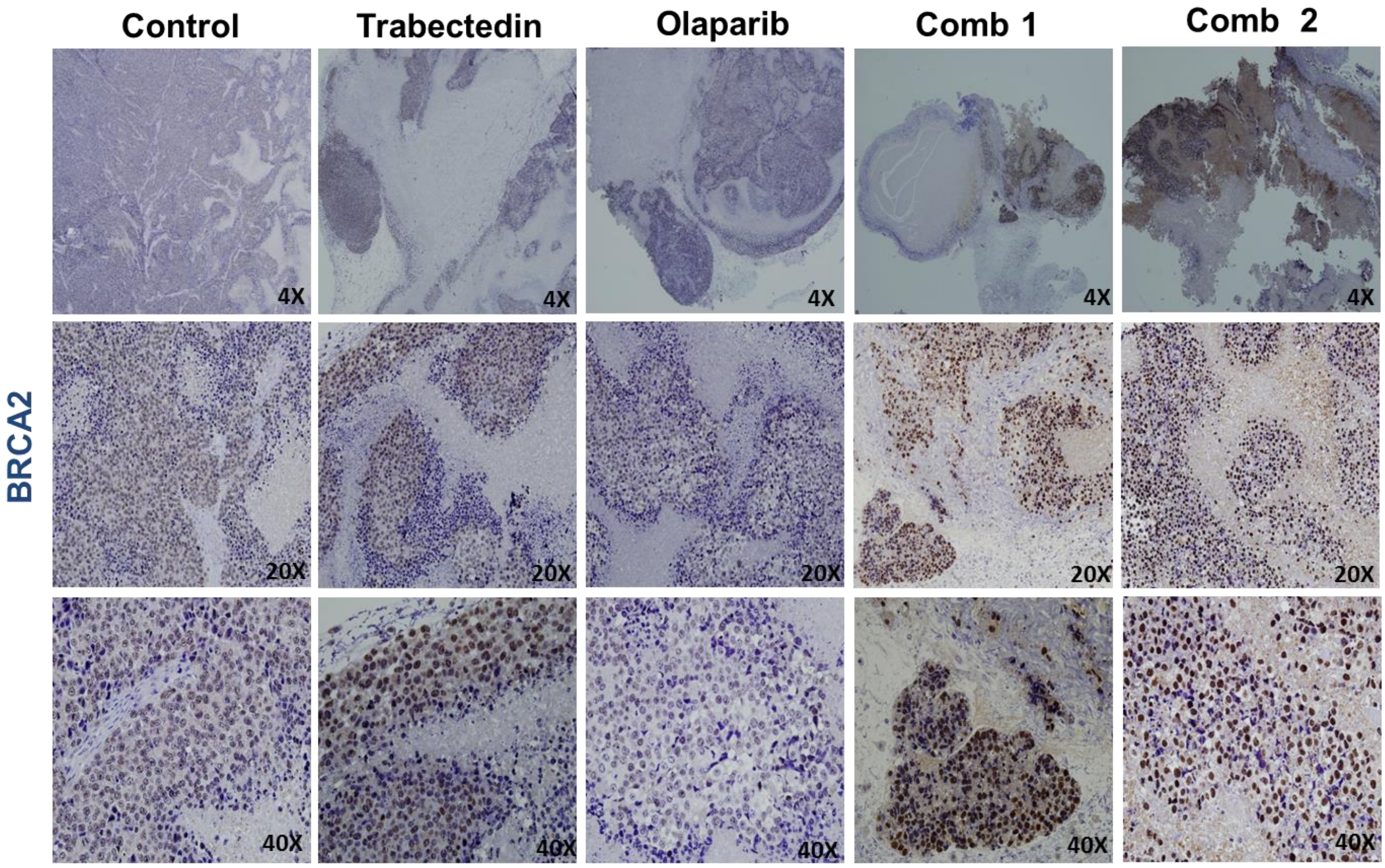

Supplementary Figure S10: BRCA2 immunostaining performed in tumor sections derived from the *in vivo* study. Comb 1 refers to Combination1. Comb 2 refers to Combination2.

Supplementary Table S1. Molecular characteristics of ES cell lines.

Cell line	Pathology	Fusion type	*TP 53* status	1q Status
**RDES**	Ewing Sarcoma	EWSR1-FLI 1, type 2	arg273stop	Gained
**WE68**	Ewing Sarcoma	EWSR1-FLI 1, type 1	Wild type	Gained
**STAET- 2.1**	Ewing Sarcoma	EWSR1-FLI 1, type 3	cys277tyr	Normal
**RM82**	Ewing Sarcoma	EWSR1-ERG	arg273his	Gained
**TC71**	Ewing Sarcoma	EWSR1-FLI 1, type 1	arg213stop	Gained
**SK-N-MC**	Ewing Sarcoma	EWSR1-FLI 1, type 1	del EX2-4	Normal
**CADO**	Ewing Sarcoma	EWSR1-ERG	Wild type	Gained
**A673**	Ewing Sarcoma	EWSR1-FLI 1, type 1	2 bp ins.codon 119	Normal
**A4573**	Ewing Sarcoma	EWSR1-FLI 1, type 3	-	Gained
**STAET -1**	Ewing Sarcoma	EWSR1-FLI 1, type 1	Wild type	Normal
**SJRH**	Rhabdomyosarcoma	-	-	-

Supplementary Table S2. Expression changes in DNA damage repair genes in ES cells from the TC71 cell line. The table depicts the most significantly up/down-expressed genes in each treatment condition. Numerical data refer to levels of expression in comparison with the controls.

Up-Regulated					
**Trabectedin**	Exp value	**Olaparib**	Exp value	**Combination**	Exp value
**BRCA1**	1.644	**EXO1**	1.927	**RAD54L**	2.548
**POLD3**	1.394	**BRIP1**	1.904	**TOP3A**	2.501
**BRIP1**	1.382	**TOP3A**	1.903	**EXO1**	2.466
**RAD18**	1.347	**RAD54L**	1.886	**BRIP1**	2.338
**NEIL3**	1.346	**RFC1**	1.700	**POLD3**	2.160
**RFC1**	1.247	**PARP3**	1.625	**RFC1**	2.159
**OGG1**	1.229	**RAD51B**	1.613	**PARP2**	1.899
**RAD52**	1.205	**XRCC1**	1.609	**BRCA1**	1.869
**XRCC1**	1.166	**POLD3**	1.597	**RAD18**	1.819
**UNG**	1.160	**RAD18**	1.550	**XRCC1**	1.810

Down-Regulated					
**Trabectedin**	Exp value	**Olaparib**	Exp value	**Combination**	Exp value
**PMS2**	-625.000	**RAD23B**	-1.302	**MSH4**	-3.358
**RAD51B**	-44.444	**NEIL2**	-1.089	**XRCC4**	-2.477
**RAD51**	-10.846	**NEIL1**	-1.046	**RAD51B**	-1.892
**MSH5**	-8.795	**DMC1**	-1.044	**MGMT**	-1.873
**TOP3B**	-8.532	**ATXN3**	-1.044	**RAD23B**	-1.423
**PMS1**	-8.019	**NTHL1**	-1.037	**ATXN3**	-1.294
**LIG1**	-5.952	**XPC**	-1.027	**SMUG1**	-1.193
**XPA**	-4.653	**PARP1**	-1.017	**NEIL2**	-1.159
**FEN1**	-4.137	**XPA**	-1.005	**APEX1**	-1.153
**XRCC6**	-4.050	**PMS1**	-1.003	**NTHL1**	-1.143

Supplementary Table S3. Expression changes in DNA damage repair genes in ES cells from the TC71 cell line. Data compared to the control (condition treated with DMSO).

		Fold Change (comparing to control group)			Gene Description
**Symbol**	**Unigene**	**Trabectedin**	**Olaparib**	**Combination**	
**APEX1**	Hs.73722	**0.64**	**1.018**	**0.8672**	APEX nuclease (multifunctional DNA repair enzyme) 1
**APEX2**	Hs.659558	**0.7434**	**1.7092**	**1.8451**	APEX nuclease (apurinic/apyrimidinic endonuclease) 2
**ATM**	Hs.367437	**0.8223**	**1.1876**	**1.069**	Ataxia telangiectasia mutated
**ATR**	Hs.271791	**0.9984**	**1.3414**	**1.3968**	Ataxia telangiectasia and Rad3 related
**ATXN3**	Hs.532632	**0.7057**	**0.9577**	**0.7729**	Ataxin 3
**BRCA1**	Hs.194143	**1.6442**	**1.4913**	**1.8691**	Breast cancer 1, early onset
**BRCA2**	Hs.34012	**1.0234**	**1.399**	**1.449**	Breast cancer 2, early onset
**BRIP1**	Hs.532799	**1.3821**	**1.9045**	**2.3385**	BRCA1 interacting protein C-terminal helicase 1
**CCNH**	Hs.292524	**0.9008**	**1.1443**	**1.2098**	Cyclin H
**CCNO**	Hs.3041	**0.6936**	**1.2828**	**1.4174**	Cyclin O
**CDK7**	Hs.184298	**0.3131**	**1.3844**	**1.6298**	Cyclin-dependent kinase 7
**DDB1**	Hs.290758	**0.3241**	**1.0567**	**1.1474**	Damage-specific DNA binding protein 1, 127kDa
**DDB2**	Hs.700338	**0.7444**	**1.2694**	**1.4446**	Damage-specific DNA binding protein 2, 48kDa
**DMC1**	Hs.339396	**0.5471**	**0.9577**	**1.4324**	DMC1 dosage suppressor of mck1 homolog, meiosis-specific homologous recombination (yeast)
**ERCC1**	Hs.435981	**0.5789**	**1.1404**	**1.2456**	Excision repair cross-complementing rodent repair deficiency, complementation group 1 (includes overlapping antisense sequence)
**ERCC2**	Hs.48	**0.859**	**1.08**	**1.0561**	Excision repair cross-complementing rodent

	7294		**32**		repair deficiency, complementation group 2
**ERCC3**	Hs.46 9872	**0.9135**	**1.14 49**	**1.1692**	Excision repair cross-complementing rodent repair deficiency, complementation group 3 (xeroderma pigmentosum group B complementing)
**ERCC4**	Hs.56 7265	**1.079**	**1.21 99**	**1.2171**	Excision repair cross-complementing rodent repair deficiency, complementation group 4
**ERCC5**	Hs.25 8429	**1.0688**	**1.13 8**	**1.3476**	Excision repair cross-complementing rodent repair deficiency, complementation group 5
**ERCC6**	Hs.65 4449	**0.7418**	**1.35 66**	**1.1502**	Excision repair cross-complementing rodent repair deficiency, complementation group 6
**ERCC8**	Hs.43 5237	**0.703**	**1.05 84**	**0.9856**	Excision repair cross-complementing rodent repair deficiency, complementation group 8
**EXO1**	Hs.49 8248	**0.6029**	**1.92 73**	**2.4661**	Exonuclease 1
**FEN1**	Hs.40 9065	**0.2417**	**1.28 77**	**1.7279**	Flap structure-specific endonuclease 1
**LIG1**	Hs.17 70	**0.168**	**1.02 5**	**1.1166**	Ligase I, DNA, ATP-dependent
**LIG3**	Hs.10 0299	**0.5081**	**1.25 77**	**0.9574**	Ligase III, DNA, ATP-dependent
**LIG4**	Hs.16 6091	**0.8789**	**1.49 08**	**1.6593**	Ligase IV, DNA, ATP-dependent
**MGMT**	Hs.50 1522	**0.568**	**1.05 37**	**0.534**	O-6-methylguanine-DNA methyltransferase
**MLH1**	Hs.19 5364	**1.0108**	**1.04 15**	**0.9544**	MutL homolog 1, colon cancer, nonpolyposis type 2 (E. coli)
**MLH3**	Hs.43 6650	**1.0298**	**1.48 32**	**1.2214**	MutL homolog 3 (E. coli)
**MMS19**	Hs.50 0721	**0.8353**	**1.22 65**	**1.2222**	MMS19 nucleotide excision repair homolog (S. cerevisiae)
**MPG**	Hs.45 9596	**0.8467**	**1.19 12**	**1.0777**	N-methylpurine-DNA glycosylase
**MRE11 A**	Hs.19 2649	**0.8344**	**1.08 33**	**1.236**	MRE11 meiotic recombination 11 homolog A (S. cerevisiae)
**MSH2**	Hs.59 7656	**0.7699**	**1.23 99**	**1.4563**	MutS homolog 2, colon cancer, nonpolyposis type 1 (E. coli)
**MSH3**	Hs.28 0987	**0.321**	**1.31 93**	**0.9042**	MutS homolog 3 (E. coli)
**MSH4**	Hs.21 6639	**0.3384**	**1.23 4**	**0.2978**	MutS homolog 4 (E. coli)

**MSH5**	Hs.64 7011	**0.1137**	**1.49 45**	**1.4797**	MutS homolog 5 (E. coli)
**MSH6**	Hs.44 5052	**0.7723**	**1.04 11**	**1.1494**	MutS homolog 6 (E. coli)
**MUTY H**	Hs.27 1353	**0.7336**	**1.15 51**	**1.1608**	MutY homolog (E. coli)
**NEIL1**	Hs.51 2732	**0.816**	**0.95 57**	**0.8762**	Nei endonuclease VIII-like 1 (E. coli)
**NEIL2**	Hs.29 3818	**0.6822**	**0.91 84**	**0.8625**	Nei endonuclease VIII-like 2 (E. coli)
**NEIL3**	Hs.40 5467	**1.3464**	**1.11 8**	**1.2745**	Nei endonuclease VIII-like 3 (E. coli)
**NTHL1**	Hs.66 196	**0.3407**	**0.96 4**	**0.875**	Nth endonuclease III-like 1 (E. coli)
**OGG1**	Hs.38 0271	**1.2294**	**1.20 9**	**1.0776**	8-oxoguanine DNA glycosylase
**PARP1**	Hs.17 7766	**0.5906**	**0.98 34**	**0.9337**	Poly (ADP-ribose) polymerase 1
**PARP2**	Hs.40 9412	**0.711**	**1.16 88**	**1.8986**	Poly (ADP-ribose) polymerase 2
**PARP3**	Hs.27 1742	**0.4062**	**1.62 48**	**1.4864**	Poly (ADP-ribose) polymerase family, member 3
**PMS1**	Hs.11 1749	**0.1247**	**0.99 75**	**0.9253**	PMS1 postmeiotic segregation increased 1 (S. cerevisiae)
**PMS2**	Hs.63 2637	**0.0016**	**1.07 41**	**1.0127**	PMS2 postmeiotic segregation increased 2 (S. cerevisiae)
**PNKP**	Hs.78 016	**0.9887**	**1.31 08**	**1.3773**	Polynucleotide kinase 3'-phosphatase
**POLB**	Hs.65 4484	**1.123**	**1.15 87**	**1.3479**	Polymerase (DNA directed), beta
**POLD3**	Hs.82 502	**1.3943**	**1.59 69**	**2.1602**	Polymerase (DNA-directed), delta 3, accessory subunit
**POLL**	Hs.52 3230	**0.2838**	**1.14 24**	**1.3329**	Polymerase (DNA directed), lambda
**PRKD C**	Hs.49 1682	**1.1391**	**1.32 54**	**1.1871**	Protein kinase, DNA-activated, catalytic polypeptide
**RAD18**	Hs.37 5684	**1.3467**	**1.55 02**	**1.8189**	RAD18 homolog (S. cerevisiae)
**RAD21**	Hs.81 848	**0.7353**	**1.02 63**	**0.9866**	RAD21 homolog (S. pombe)
**RAD23**	Hs.64	**0.4415**	**1.12**	**1.3637**	RAD23 homolog A (S. cerevisiae)

**A**	3267		**59**		
**RAD23 B**	Hs.52 1640	**0.4044**	**0.76 79**	**0.7029**	RAD23 homolog B (S. cerevisiae)
**RAD50**	Hs.65 5835	**0.5595**	**1.34 4**	**1.1334**	RAD50 homolog (S. cerevisiae)
**RAD51**	Hs.63 1709	**0.0922**	**1.03 08**	**1.2539**	RAD51 homolog (S. cerevisiae)
**RAD51 B**	Hs.17 2587	**0.0225**	**1.61 29**	**0.5286**	RAD51 homolog B (S. cerevisiae)
**RAD51 C**	Hs.41 2587	**0.5584**	**1.13 62**	**1.1026**	RAD51 homolog C (S. cerevisiae)
**RAD51 D**	Hs.63 1757	**0.9553**	**1.11 58**	**0.9528**	RAD51 homolog D (S. cerevisiae)
**RAD52**	Hs.70 9202	**1.2049**	**1.25 17**	**1.4908**	RAD52 homolog (S. cerevisiae)
**RAD54 L**	Hs.64 2042	**0.6544**	**1.88 58**	**2.5478**	RAD54-like (S. cerevisiae)
**RFC1**	Hs.50 7475	**1.2467**	**1.70 01**	**2.1594**	Replication factor C (activator 1) 1, 145kDa
**RPA1**	Hs.46 1925	**0.8569**	**1.01 46**	**1.0292**	Replication protein A1, 70kDa
**RPA3**	Hs.48 7540	**0.6118**	**1.02 5**	**1.1033**	Replication protein A3, 14kDa
**SLK**	Hs.59 1922	**0.4988**	**1.15 78**	**1.2302**	STE20-like kinase
**SMUG 1**	Hs.63 2721	**0.463**	**1.13 86**	**0.8381**	Single-strand-selective monofunctional uracil-DNA glycosylase 1
**TDG**	Hs.58 4809	**0.8917**	**1.09 34**	**1.1476**	Thymine-DNA glycosylase
**TOP3A**	Hs.59 2115	**0.3991**	**1.90 27**	**2.5013**	Topoisomerase (DNA) III alpha
**TOP3B**	Hs.43 6401	**0.1172**	**1.39 49**	**1.5978**	Topoisomerase (DNA) III beta
**TREX1**	Hs.70 7026	**0.8717**	**1.49 47**	**1.6449**	Three prime repair exonuclease 1
**UNG**	Hs.19 1334	**1.1605**	**1.16 64**	**1.5278**	Uracil-DNA glycosylase
**XAB2**	Hs.98 22	**1.126**	**1.32 67**	**1.6688**	XPA binding protein 2
**XPA**	Hs.65 4364	**0.2149**	**0.99 54**	**1.1093**	Xeroderma pigmentosum, complementation group A

**XPC**	Hs.47<br>5538	**0.4052**	**0.97<br>39**	**0.973**	Xeroderma pigmentosum, complementation group C
**XRCC1**	Hs.98<br>493	**1.1662**	**1.60<br>94**	**1.8095**	X-ray repair complementing defective repair in Chinese hamster cells 1
**XRCC2**	Hs.64<br>7093	**0.9138**	**1.38<br>56**	**1.2881**	X-ray repair complementing defective repair in Chinese hamster cells 2
**XRCC3**	Hs.59<br>2325	**0.3498**	**1.03<br>26**	**1.143**	X-ray repair complementing defective repair in Chinese hamster cells 3
**XRCC4**	Hs.56<br>7359	**0.33**	**1.04<br>01**	**0.4037**	X-ray repair complementing defective repair in Chinese hamster cells 4
**XRCC5**	Hs.38<br>8739	**0.5948**	**1.20<br>94**	**1.1488**	X-ray repair complementing defective repair in Chinese hamster cells 5 (double-strand-break rejoining)
**XRCC6**	Hs.29<br>2493	**0.2469**	**1.14<br>74**	**1.085**	X-ray repair complementing defective repair in Chinese hamster cells 6
**XRCC6<br>BP1**	Hs.61<br>188	**0.2786**	**1.25<br>61**	**1.286**	XRCC6 binding protein 1

Supplementary Table S4. Top pathways covered by the array. Major pathways affected after treatment with the combination of Olaparib and Trabectedin in ES cells from the TC71 cell line are involved in the repair of DSB. The ratio indicates the number of affected genes of a pathway/total number of genes of a pathway. The p-value is an estimation of the likelihood that the correlation between the number of affected genes in our study that participate in a pathway and the total number of genes that are known to be associated with that pathway will be due to random association. p-values<0.05 indicate statistically significant differences.

Pathways	Ratio	p-value
**NER**	14/35 (0.4)	1.02E-23
**NHEJ**	11/16 (0.688)	1.81E-23
**HR**	9/16 (0.562)	4.41E-18
**BRCA1**	13/63 (0.206)	4.52E-18
**HBCS**	15/119 (0.126)	2.24E-17
